# The E3 ligase NEURL3 suppresses epithelial-mesenchymal transition and metastasis in nasopharyngeal carcinoma by promoting vimentin degradation

**DOI:** 10.1186/s13046-024-02945-9

**Published:** 2024-01-09

**Authors:** Shi-Qing Zhou, Ping Feng, Ming-Liang Ye, Sheng-Yan Huang, Shi-Wei He, Xun-Hua Zhu, Jun Chen, Qun Zhang, Ying-Qing Li

**Affiliations:** 1 State Key Laboratory of Oncology in South China, Guangdong Key Laboratory of Nasopharyngeal Carcinoma Diagnosis and Therapy, Guangdong Provincial Clinical Research Center for Cancer, Sun Yat-sen University Cancer Center, 651 Dongfeng Road East, Guangzhou, 510060 People’s Republic of China; 2 Otorhinolaryngology Head and Neck Department, The Second Affiliated Hospital of Guangzhou University of Chinese Medicine, Guangzhou, 510120 China; 3 Department of Radiation Oncology, The First Affiliated Hospital of Sun Yat-sen University, 58 Zhongshan Second Road, Guangzhou, 510080 People’s Republic of China

**Keywords:** Nasopharyngeal carcinoma, NEURL3, Vimentin, Metastasis, DNA methylation

## Abstract

**Background:**

Metastasis has emerged as the major reason of treatment failure and mortality in patients with nasopharyngeal carcinoma (NPC). Growing evidence links abnormal DNA methylation to the initiation and progression of NPC. However, the precise regulatory mechanism behind these processes remains poorly understood.

**Methods:**

Bisulfite pyrosequencing, RT-qPCR, western blot, and immunohistochemistry were used to test the methylation and expression level of NEURL3 and its clinical significance. The biological function of NEURL3 was examined both in vitro and in vivo. Mass spectrometry, co-immunohistochemistry, immunofluorescence staining, and ubiquitin assays were performed to explore the regulatory mechanism of NEURL3.

**Results:**

The promoter region of *NEURL3*, encoding an E3 ubiquitin ligase, was obviously hypermethylated, leading to its downregulated expression in NPC. Clinically, NPC patients with a low NEURL3 expression indicated an unfavorable prognosis and were prone to develop distant metastasis. Overexpression of NEURL3 could suppress the epithelial mesenchymal transition and metastasis of NPC cells in vitro and in vivo. Mechanistically, NEURL3 promoted Vimentin degradation by increasing its K48-linked polyubiquitination at lysine 97. Specifically, the restoration of Vimentin expression could fully reverse the tumor suppressive effect of NEURL3 overexpression in NPC cells.

**Conclusions:**

Collectively, our study uncovers a novel mechanism by which NEURL3 inhibits NPC metastasis, thereby providing a promising therapeutic target for NPC treatment.

**Supplementary Information:**

The online version contains supplementary material available at 10.1186/s13046-024-02945-9.

## Background

Nasopharyngeal carcinoma (NPC) is a malignant tumor originating from nasopharyngeal epithelium and displays a marked geographic heterogeneity, with a prominent prevalence in South China, Southeastern Asia, and North Africa [1, 2]. This unique distribution is attributed to the genetic susceptibility, dietary and infection factors, and so on [3, 4]. The application of intensity-modulated radiation therapy (IMRT) and its combination with chemotherapy have remarkably improved the locoregional control of this disease, and distant metastasis becomes the major reason of treatment failure and cancer-related mortality of NPC [5–7]. Therefore, there is an urgent need to better explore the regulatory mechanism underlying NPC metastasis and guide a more personalized therapy for NPC patients.

Whole genome and exome sequencing reveals that genomic alterations, such as mutation, deletion, amplification, are infrequent in NPC [8–10]. As a kind of epigenetic modification, DNA methylation is considered to be a stone characteristic of NPC [11, 12]. A genome-wide DNA methylation study from a HongKong research group demonstrates that DNA methylation dysregulation is a very common phenotype in several types of tumors, which is most typical in NPC [13]. Importantly, over 90% of differentially methylated CpG sites are hypermethylated in NPC [13], and the promoter hypermethylation of several tumor suppressors is associated with poor prognosis of NPC patients [14]. Additionally, several tumor suppressors, including SFRP1, HOPX, SHISA3, and USP44, are reported to be silenced by DNA methylation and be involved in regulating NPC metastasis and treatment resistance [15–18], suggesting that DNA methylation dysregulation is an essential event in NPC.

The ubiquitin-proteasome system plays a pivotal role in regulating protein degradation, functionality, and subcellular trafficking to maintain cellular homeostasis [19]. In this process, the E3 ubiquitin ligases confer specificity to the substrates that undergo ubiquitination, thereby endowing E3 with the ability to regulate the localization, activity, interaction, and abundance of nearly all cellular proteins and exert influence over various aspects of cell biology [20, 21]. Aberrant modulation of E3 ligases activity has profound implications in diverse pathological conditions, particularly with regard to tumorigenesis [21, 22]. The aberrant expression of E3 ligases leads to uncontrolled cell proliferation and immortalization, epithelial-mesenchymal transition (EMT), tumor invasion and metastasis [23, 24]. Recent studies have also revealed a significant association between DNA hypermethylation and E3 ligases, resulting in increased radioresistance and enhanced tumor metastasis in NPC [17, 18].

Here, we identified that neuralized E3 ubiquitin protein ligase 3 (NEURL3) was highly methylated in NPC based on the integrated analysis of two genome-wide DNA methylation microarray datasets [13, 14]. NEURL3 is initially characterized in mouse alveolar epithelial cells following lipopolysaccharide induction [25, 26], and it can regulate host antiviral immune, pulmonary embryogenesis and spermatogenesis [27–30]. However, the exact regulatory role in tumorigenesis and progression is still not clear. We found that the downregulation of NEURL3 was attributed to its promoter hypermethylation and indicated poor prognosis in NPC patients. Overexpression of NEURL3 suppressed NPC cell EMT, migration, invasion, and metastasis by promoting the degradation of Vimentin in a ubiquitin-proteasome pathway. Collectively, our findings emphasize the vital role and clinical significance of NEURL3-Vimentin axis in NPC and provide potential therapeutic targets for NPC.

## Materials and methods

### Public datasets and bioinformatics analysis

Two genome-wide DNA methylation microarray datasets (GSE52068 and GSE62336) were integrated to identify differentially methylated genes between normal nasopharynx and NPC tissues [13, 14]. A genome-wide gene expression dataset (GSE102349) was downloaded and then the 113 NPC cases were divided into high or low NEURL3 expression groups using the X-tile software [31]. Gene set enrichment analysis (GSEA) was conducted using the GSEA v4.2.3 software (https://www.gsea-msigdb.org/gsea/msigdb/index.jsp) to identify significantly enriched gene sets that related to NEURL3 expression level.

### Clinical samples

A total of 23 fresh-frozen NPC and 23 normal nasopharynx tissue samples were collected for DNA methylation and expression analysis from the Sun Yat-sen University Cancer Center (Guangzhou, China). Another 212 paraffin-embedded NPC tissue samples were obtained for prognostic analysis from the Sun Yat-sen University Cancer Center between Jan 2004 and Jan 2007. No patients had received any antitumor therapy before biopsy. The clinical features of NPC patients are listed in Supplementary Table 1. This study was approved by the Institutional Ethical Review Boards of the Sun Yat-sen University Cancer Center, and the requirement of informed consent was waived (No. B2023-001-01).

### Cell culture and treatment

A list of 9 NPC cell lines (HONE1, HK1, SUNE1, CNE1, CNE2, S18, S26, 5-8 F, 6-10B) were maintained in RPMI-1640 medium (Gibco, NY, USA) contained 10% fetal bovine serum (FBS; ExCell Bio, Taicang, China). The normal nasopharyngeal epithelial cell line NP69 was cultured in keratinocyte serum-free medium (KSFM; Gibco). 293T cells were cultured in DMEM (Gibco) contained 10% FBS. All cell lines were authenticated by short tandem repeat profiling and tested for mycoplasma contamination. Cells were treated with 10 µM MG132 (Selleck, TX, USA) or 50 µM chloroquine (CQ; Sigma-Aldrich, MO, USA) for 8 h or 100 µg/mL cycloheximide (CHX; Sigma-Aldrich) for 0, 12, 24, 36, or 48 h.

### Plasmid construction and transfection

The coding sequences (CDS) of NEURL3 and Vimentin that tagged with HA, FLAG, or Myc were separately cloned into pSin-EF2-puro vector to generate overexpression plasmids: pSin-EF2-puro-NEURL3-HA, pSin-EF2-puro-NEURL3-Myc, and pSin-EF2-puro-Vimentin- FLAG. The shNEURL3 sequences were designed by Invitrogen Block-iT RNAi Designer, and then synthesized and inserted into pLKO.1-RFP vector to construct pLKO.1-shNEURL3 #1/2 plasmids. The shNEURL3 sequences are shown in Supplementary Table 2.

For transient transfection, cells were transfected with overexpression or shRNA plasmids using a DNA transfection reagent (Neofect, Beijing, China) according to the manufacturer’s instructions, and then collected for further experiments after 24 ~ 48 h. For stable transfection, the lentiviral packaging plasmids pSPAX2 and pMD2G were co-transfected with either the NEURL3 overexpression plasmid or an empty plasmid into 293T cells to generate virus supernatant. Subsequently, cells were infected with the virus supernatant for a period of 48 h, and then selected with puromycin (Invitrogen, NY, USA). RT-qPCR and western blotting assays were used to assess the transfection efficiency.

### Bisulfite pyrosequencing (BSP)

DNA was isolated from fresh-frozen tissues and cell lines using AllPrep RNA/DNA Mini Kit (Qiagen) or EZ1 DNA Tissue Kit (Qiagen), and then subjected to bisulfite modification by EpiTect Bisulfite Kit (Qiagen). The primers for bisulfite pyrosequencing were designed using the PyroMark Assay Design Software 2.0 (Qiagen), and the primer sequences for PCR and sequencing are listed in Supplementary Table 2. The quantification of NERUL3 methylation levels was performed on the PyroMark Q96 ID System (Qiagen).

### RT-qPCR

Total RNA was isolated by the TRIzol reagent (Invitrogen), and then subjected to reverse transcription reaction using random primers (Promega, Madison, WI, USA) and M-MLV reverse transcriptase (Promega). Quantitative PCR was conducted using the SYBR Green PCR Master Mix (Applied Biosystems; Thermo Fisher Scientific, MA, USA) on a CFX96 real-time system (Bio-Rad, CA, USA). GAPDH was used as the internal control. All of the primer sequences are showed in Supplementary Table 2.

### Western blot

Total protein was isolated using RIPA buffer. Equal amounts (20 ~ 50 µg) of proteins were separated by SDA-PAGE and transferred to PVDF membranes (Merck Millipore, MA, USA). After being blocked with a 5% BSA solution, the membranes were incubated overnight at 4 °C with the following primary antibodies: anti-NEURL3 (1:1000, 16648-1-AP; Proteintech, Wuhan, China), anti-Vimentin (1:1000, 5741 S; Cell Signaling Technology, MA, USA), anti-E-cadherin (1:1000, 3195 S; Cell Signaling Technology), or anti-β-actin (1:5000, 66009-1-Ig; Proteintech). Then, the membranes were incubated with goat anti-mouse or anti-rabbit antibody (Cell Signaling Technology) at room temperature for 1 h. Protein bands were visualized using an enhanced chemiluminescence.

### Immunohistochemistry

The immunohistochemistry was performed as previously described [16–18]. Briefly, the sections were first deparaffinized and rehydrated, and the endogenous peroxidase activity was blocked. Sections were then subjected to microwave antigenic retrieval, and the non-specific binding was blocked. Finally, sections were incubated with anti-NEURL3 antibody (1:200, 16648-1-AP; Proteintech) overnight at 4 °C, followed by biotinylated secondary antibody (Boster, Wuhan, China). All of the sections were scored by two pathologists according to the immunoreactive score (IRS) system as previously described [16–18].

### Cell viability and colony formation assays

For the cell viability assay, cells were seeded into 96-well plates. At specific time points (0, 1, 2, 3, 4, or 5 days), 10 µl of Cell Counting Kit (CCK-8; TargetMol, MA, USA) was added to each well, and the absorbance at 450 nm was measured using a spectrophotometer 2 h later. For the clone formation assay, cells were seeded into 6-well plates. After 7 ~ 10 days, the plates were washed, fixed, and stained, and the colonies were counted.

### Wound healing, transwell migration and invasion assays

For the wound healing assay, cells were seeded into 6-well plates and cultured to reach almost confluence. A straight line was drawn along the diameter of each well using a sterile 10 µl tip. The culture medium was replaced with serum-free medium, and images were acquired at specific time points (0, 12, or 24 h) by an inverted microscope. The migration and invasion assays were conducted using Transwell Chambers (Corning, NY, USA) pre-coated without or with Matrigel (BD Biosciences, CA, USA). Cells were suspended in serum-free medium and seeded into the upper chamber. The lower chamber was filled with medium supplemented with 10% FBS. Following an incubation period of 12 ~ 16 h, cells were fixed, stained, and counted under an inverted microscope (NIKON Eclipse Ti2-U).

### Immunofluorescence (IF) staining

Cells were seeded into 24-well plates coated with sterile coverslips, fixed, permeabilized with 0.5% Triton X-100, and incubated with Actin-Tracker Green (1:100; Beyotime, Shanghai, China), anti-NEURL3 (1:100, 16648-1-AP; Proteintech), anti-Vimentin (1:100, 5741 S; Cell Signaling Technology) or anti-E-cadherin (1:250, 3195 S; Cell Signaling Technology) antibody. Cells were incubated with secondary antibody, and the nuclei were counterstained with DAPI (Sigma-Aldrich). Images were acquired by a Laser Confocal Microscope (LSM880; Zeiss, Oberkochen, Germany).

### Co-immunoprecipitation (co-IP) and mass spectrometry

Cells were lysed on ice by IP lysis buffer, sonicated, and then centrifugated to collect the supernatant as previously described [16–18]. The protein supernatant was incubated with 2 µg of indicated antibodies or IgG at 4 °C overnight. The immune complexes were incubated with Pierce Protein A/G magnetic beads (Thermo Fisher Scientific) and then washed with IP wash buffer. The immune complexes were denatured, separated by SDS-PAGE, and stained by silver staining. The differentially protein bands were analyzed by mass spectrometry (Weifei Biotechnology Co., Shenzhen, China), and proteins of interest were detected by western blot. The antibodies used for co-IP included anti-HA-tag (H6908, Sigma-Aldrich), anti-Flag-tag (F1804, Sigma-Aldrich), and anti-Vimentin (5741 S, Cell Signaling Technology).

### Ubiquitin assay

All ubiquitin assays were performed in a denaturing condition as previously described [17, 18]. Cells were lysed on ice in IP lysis buffer, and the cell lysates were denatured at 95 °C for 5 min. Co-IP was performed with specific antibodies as mentioned above, and the lysates and immune complexes were subjected for western blot analysis.

### Animal models

All procedures were in accordance with the guidelines of the Institutional Animal Care and Use Committee of Sun Yat-sen University Cancer Center (L025501202208009). BALB/c nude mice (4 ~ 5 weeks old, female) were obtained from the Animal Facility of the Sun Yat-sen University Cancer Center. 3 × 10^5^ or 1 × 10^6^ SUNE1 cells that stably overexpressed NEURL3 or empty vector were injected into footpad or tail vein to establish lymph node metastatic model (*n* = 8 per group) or lung metastatic colonization model (*n* = 8 per group). After 6 ~ 8 weeks, mice were sacrificed, and the footpad tumors, the inguinal lymph nodes, and the lung tissues were excised, fixed, embedded, and sliced for H&E staining and immunohistochemistry (IHC) with an anti-pan-cytokeratin antibody to quantify the metastatic ratio of inguinal lymph node and metastatic tumor nodules in lung tissues as previously described [32].

### Statistical analysis

Data are presented as the mean ± standard deviation (SD) of three independent duplicate experiments. Difference between groups was compared with Student’s *t*-test, chi-square test, or Fisher’s exact test. The Kaplan-Meier method was used to estimate survival probabilities, and the long-rank test was used to compare survival curves. Multivariate COX regression analysis was used to test independent prognostic factors. Statistical analyses were conducted using GraphPad Prism version 8.0.1 software. All tests were two-tailed, and a *p* < 0.05 was considered statistically significant.

## Results

### NEURL3 is hypermethylated in NPC

**Fig. 1:**
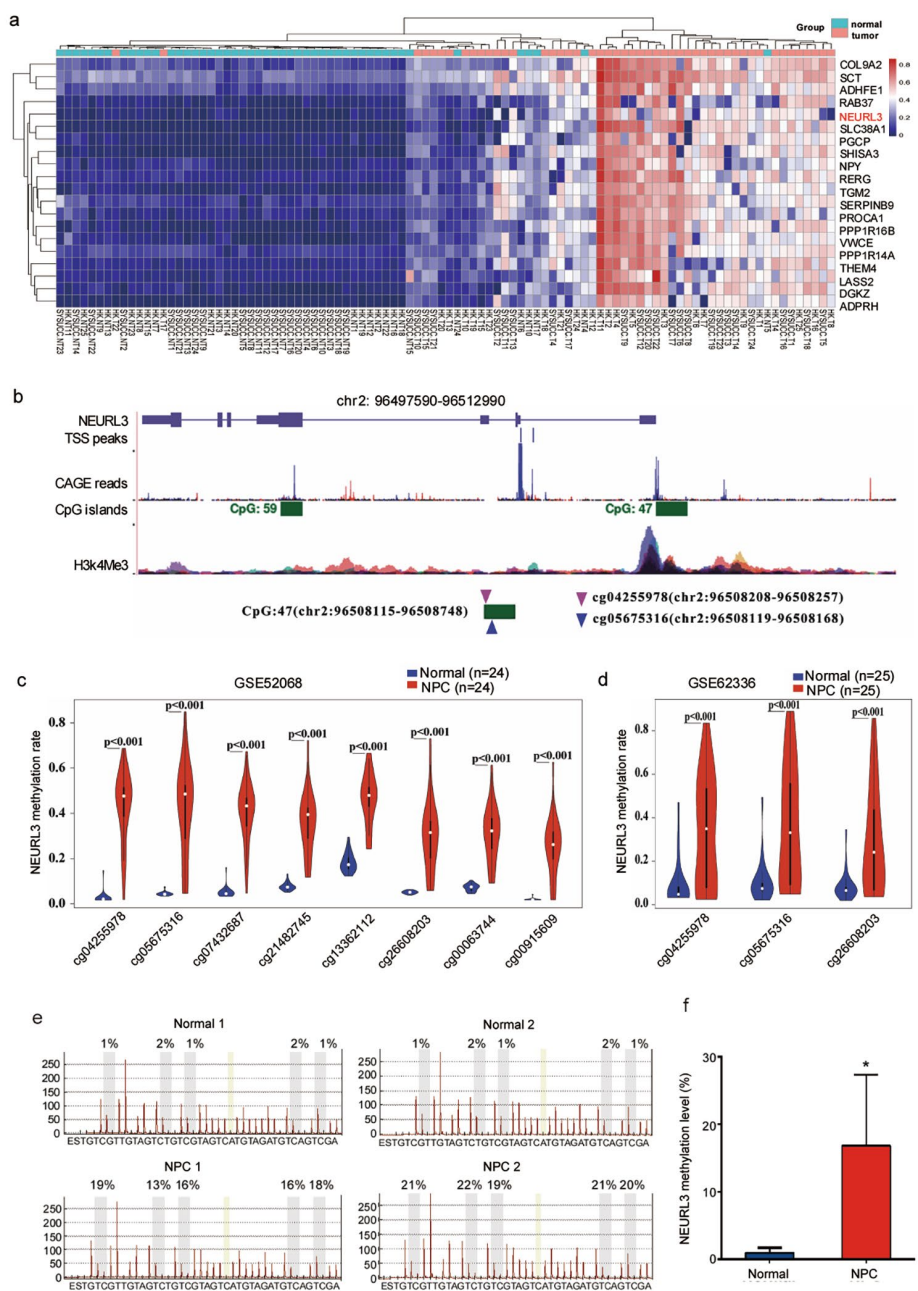
NEURL3 is hypermethylated in NPC. **(a)** Heatmap clustering of the top 20 hypermethylated genes based on integrated analysis of two genome-wide DNA methylation microarray datasets (GSE52068 and GSE62336) in NPC and normal nasopharyngeal tissues. **(b)** The genome features of NEURL3 observed using the UCSC genome browser and schematic diagram of the CpG island sites at NEURL3 promoter region. **(c)** Methylation levels of NEURL3 in NPC tissues (*n* = 24) and normal tissues (*n* = 24) in the methylation microarray dataset GSE52068. **(d)** Methylation levels of NEURL3 in NPC tissues (*n* = 25) and normal tissues (*n* = 25) in HongKong methylation microarray dataset GSE62336. **(e)** Representative images of bisulfite pyrosequencing analysis of the NEURL3 promoter region. **(f)** Statistical analysis of methylation levels of NEURL3 in NPC (*n* = 8) and normal (*n* = 8) tissues. Data in c, d, and f are presented as the mean ± SD, and the *p*-values were determined by Student’s *t*-test (*p < 0.05)

Through integrated analysis of two genome-wide DNA methylation microarray datasets [13, 14], we found the top 20 hypermethylated genes in NPC (*n* = 49) compared with the normal nasopharyngeal tissue (*n* = 49) samples (Fig. 1a). Among them, we selected NEURL3, the most changed E3 ubiquitin ligase, for further analysis. Two CpG islands (CpG47 and CpG59) were identified in the NEURL3 gene based on USCS Genome Browder. By analyzing of ENCODE database, we revealed a strong enrichment of H3K4me3, a kind of histone modification, in the CpG47 island of NEURL3, demonstrating that the CpG47 island of NEURL3 might serve as an active promoter. Then, cap analysis gene expression (CAGE) peaks that are corresponded to transcription start sites (TSSs) were identified in the downstream of the CpG47 island of NEURL3 by the FANTOM project (Fig. 1b).

Based on a previous microarray data (GSE52068), we found 8 CpG sites of the NEURL3 gene that were significantly hypermethylated in NPC compared with the normal nasopharynx tissues (Fig. 1c). Through analysis of the Hong Kong microarray dataset (GSE62366), three of the eight CpG sites (cg04255978, cg05675316, and cg26608203) were verified to be markedly hypermethylated in NPC tissues (Fig. 1d). We then tested the methylation level of cg04255978, the most significantly methylated CpG site in NEURL3 gene, in another 8 NPC and 8 normal nasopharyngeal tissues by pyrophosphate sequencing, and found that the methylation level of cg04255978 was significantly increased in NPC tissue samples (Fig. 1e-f). These data indicate that NEURL3 is significantly hypermethylated in NPC.

### NEURL3 is downregulated and associated with poor prognosis in NPC

**Fig. 2:**
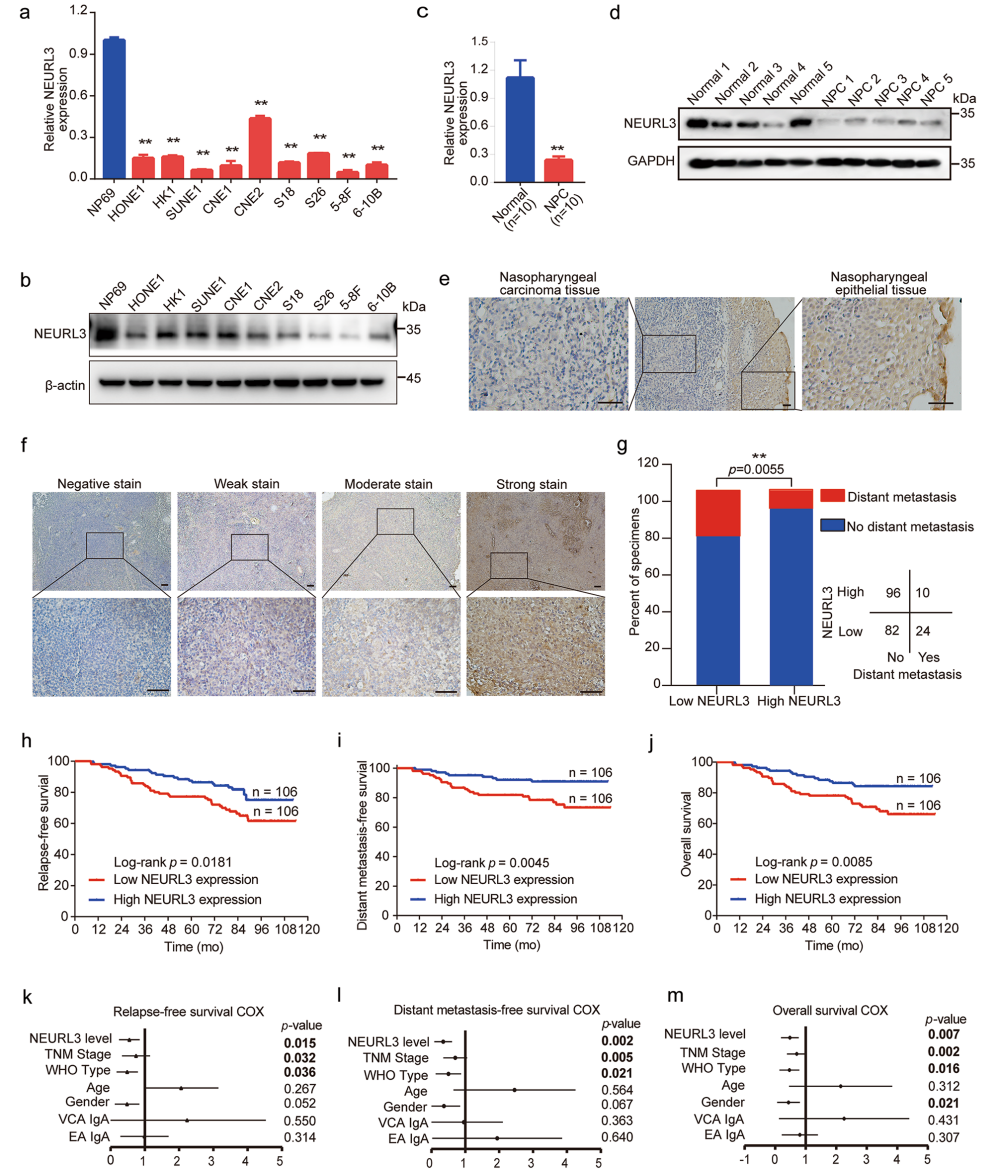
NEURL3 is downregulated and associated with poor prognosis in NPC. **(a)** Relative mRNA levels of NEURL3 in NPC cell lines and nasopharyngeal epithelial cell line NP69. **(b)** Relative protein levels of NEURL3 in NPC cell lines and nasopharyngeal epithelial cell line NP69. **(c)** Relative NEURL3 mRNA levels measured by RT-qPCR analysis in NPC (*n* = 10) and normal (*n* = 10) tissues. Data of **a** and **c** are shown as mean ± SD, and the *p*-values were determined by Student’s *t*-test (*p < 0.05). **(d)** Western blot analysis of NEURL3 protein expression in NPC (*n* = 5) and normal (*n* = 5) tissues. **(e)** Representative images displaying the NEURL3 protein levels in NPC and adjacent normal tissue determined by IHC staining. **(f)** Representative images displaying the NEURL3 protein levels in 212 NPC tissues determined by IHC staining, and the staining intensity was categorized as negative, weak, moderate, or strong stain (Scale bar, 100 μm or 20 μm). **(g)** The correlation between NEURL3 expression and posttreatment distant metastasis status. The *p*-value was determined by Chi-square test. **(h-j)** Kaplan-Meier curves were generated to evaluate relapse-free survival **(h)**, distant metastasis-free survival **(i)**, and overall survival **(j)** according to NEURL3 protein level. The *p*-values were determined by log-rank test. **(k-m)** Forest plots of multivariate Cox regression analyses were generated to show the significance of different clinical prognostic factors in NPC relapse-free survival **(k)**, distant metastasis-free survival **(l)**, and overall survival **(m)**

We then detected the expression level of NEURL3 in NPC cell lines and the immortalized nasopharyngeal epithelial cell line NP69. Compared with the NP69, we found that both the mRNA and protein levels of NEURL3 were obviously decreased in NPC cell lines (Fig. 2a-b). In addition, the mRNA and protein levels of NEURL3 were also markedly decreased in NPC tissues compared to normal nasopharyngeal tissues (Fig. 2c-e). Then, we evaluated the protein level of NEURL3 in 212 NPC samples to explore its clinical relevance by IHC staining (Fig. 2f). We divided NPC patients into a high or low NEURL3 expression group, and found that patients with a low NEURL3 expression were more prone to experience posttreatment distant metastasis (Fig. 2g). Kaplan-Meier survival analysis showed that patients with a low NEURL3 expression exhibited poorer relapse-free survival, distant metastasis-free survival, and overall survival than those with a high NEURL3 expression (Fig. 2h-j). Mulvariable Cox regression analysis indicated that NEURL3 expression level served as independent prognostic factors for NPC prognosis (Fig. 2k-m). These results demonstrate that NEURL3 is downregulated and associated with poor prognosis in NPC patients.

### NEURL3 suppresses NPC cell migration, invasion, and EMT in vitro

**Fig. 3:**
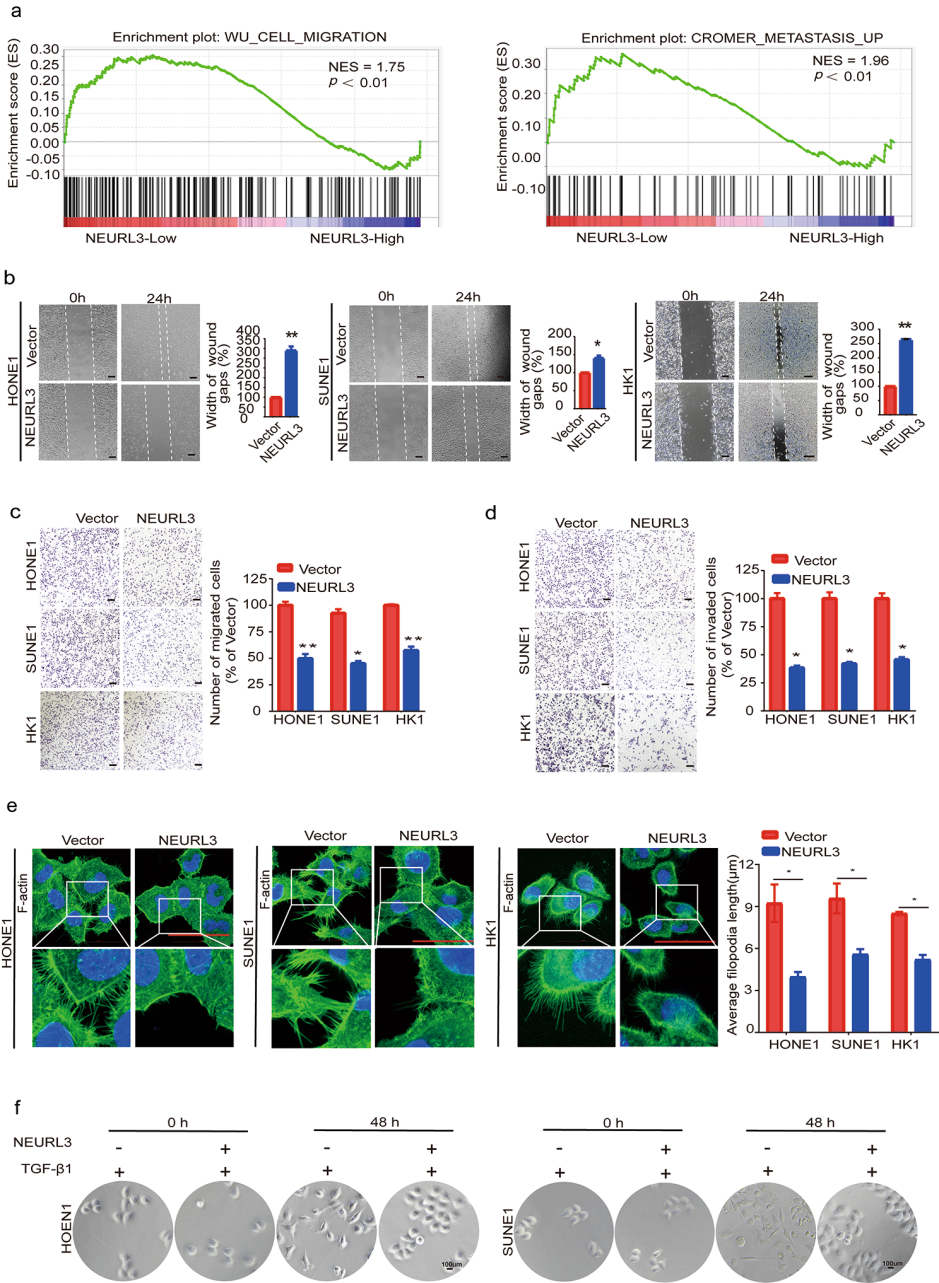
NEURL3 suppresses NPC cell migration, invasion, and EMT in vitro. **(a)** In GSEA analysis using a public RNA-seq dataset (GSE102349), the gene sets associated with cell migration and metastasis pathways were significantly enriched in NPC samples with a low NEURL3 expression. **(b)** The migratory abilities of HONE1, SUNE1, and HK1 cells transfected with HA-NEURL3 plasmid or its empty vector determined by wound healing assay. **(c-d)** The migratory **(c)** and invasive **(d)** abilities of HONE1, SUNE1, and HK1 cells transfected with HA-NEURL3 plasmid or its empty vector determined by Transwell assay. **(e)** IF staining showing the filopodia length on HONE1, SUNE1, and HK1 cells transfected with HA-NEURL3 plasmid or its empty vector. **(f)** The morphology changes induced by TGF-β1 in HONE1, and SUNE1 cells transfected with HA-NEURL3 plasmid or its empty vector. Scale bar, 50 μm. Data of **b**, **c**, **d**, and **e** are shown as mean ± SD, and the *p*-values were determined by Student’s *t*-test (*p < 0.05)

To learn the potential biological function of NEURL3 in NPC, we conducted GSEA using an RNA-seq dataset (GSE102349) containing 113 NPC samples [31]. The results showed that the gene sets associated with cell migration and metastasis were significantly enriched in NPC samples with a low NEURL3 expression (Fig. 3a). We transiently transfected NPC cell lines with NEURL3 overexpressing plasmid or its empty vector, as well as shNEURL3 plasmids or its control vector (Supplementary Fig. 1a-b), and then did in vitro functional experiments. The results of wound healing and Transwell assays showed that overexpression of NEURL3 significantly decreased the migratory and invasive abilities of NPC cells (Fig. 3b-d), while knockdown of NEURL3 enhanced the migratory and invasive abilities of NPC and NP69 cells (Supplementary Fig. 1c-d, and Fig. 2). The results of CCK8 and colony formation assays showed that overexpression of NEURL3 had no obvious effect on the NPC cell growth and proliferation (Supplementary Fig. 3). Furthermore, IF staining showed that overexpression of NEURL3 reduced the filopodia length on NPC cells, suggesting a decreased ability of cell migration and invasion (Fig. 3e). Additionally, overexpression of NEURL3 could effectively inhibit TGF-β1-induced EMT phenotype of NPC cells (Fig. 3f). These findings suggest that NEURL3 suppresses NPC cell migration, invasion and EMT in vitro.

### NEURL3 interacts with vimentin to promote its degradation

**Fig. 4:**
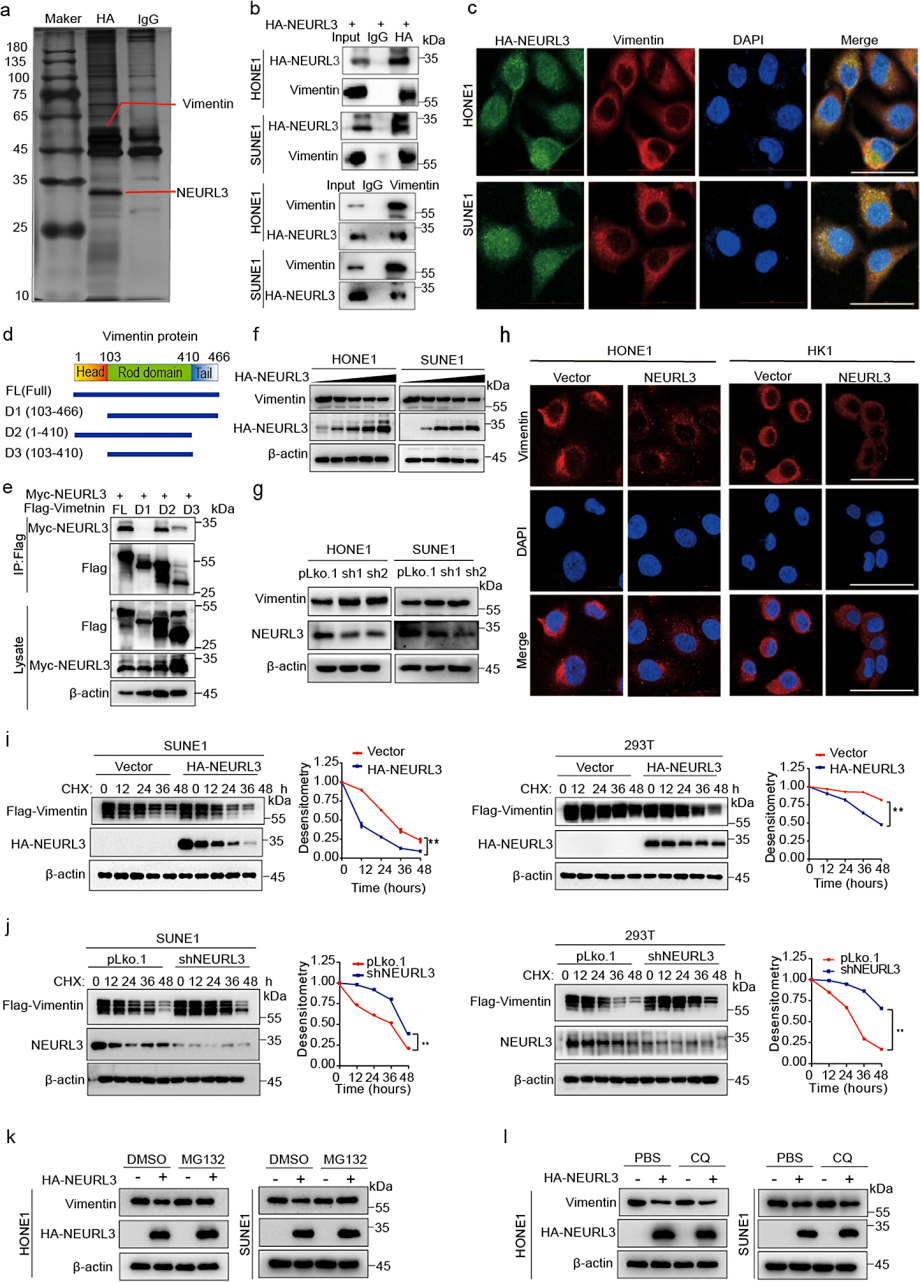
NEURL3 interacts with Vimentin to promote its degradation. **(a)** Silver staining of SDS-PAGE gels showing HA immunoprecipitates that were pulled down from SUNE1 cells transfected with HA-NEURL3 plasmid. The interest proteins are indicated. **(b)** Co-IP with anti-HA or Vimentin antibodies showing the interactions between HA-NEURL3 and endogenous Vimentin in HONE1 and SUNE1 cells. **(c)** IF staining showing co-localization of exogenous HA-NEURL3 and endogenous Vimentin in HONE1 and SUNE1 cells (Scale, 50 μm). **(d)** Schematic diagram showing the structure of Vimentin or its deletion mutant plasmids. **(e)** Co-IP revealing interactions of NEURL3 with different Vimentin mutants in 293T cells. **(f-g)** Vimentin protein levels in HONE1 and SUNE1 cells transfected with gradient concentrations of HA-tagged NEURL3 plasmid **(f)**, as well as shNEURL3 plasmids or its control vector **(g)**. **(h)** IF staining showing Vimentin protein levels in HONE1 and HK1 cells transfected with HA-NEURL3 plasmid or its vector control (Scale bar, 50 µM). **(i-j)** Representative images and greyscale analyses of Vimentin protein levels in SUNE1 and 293T cells transfected with HA-NEURL3 plasmid or its vector control **(i)**, as well as shNEURL3 plasmid or its control vector (**j**), after the CHX treatment. **(k-l)** Vimentin protein levels in HONE1 and SUNE1 cells transfected with HA-NEURL3 plasmid or its vector control after treated with MG132 **(k)** and CQ **(l)**. Data of **i** and **j** are shown as mean ± SD, and the *p*-values were determined by Student’s *t*-test (*p < 0.05)

To clarify the molecular mechanism by which NEURL3 inhibits NPC migration, invasion and EMT, we performed mass spectrometry analysis and identified Vimentin, which is one of the EMT-associated markers, as a potential target of NEURL3 (Fig. 4a; Supplementary Fig. 4a; Supplementary Table 3). Co-IP confirmed the interactions between HA-NEURL3 and endogenous Vimentin (Fig. 4b), and these interactions were confirmed by IF staining, showing the co-localization of these two proteins in the cytoplasm of NPC cells (Fig. 4c). To determine the specific domain(s) of Vimentin interacting with NEURL3, we first generated Flag-tagged Vimentin (FL) and three deletion mutant plasmids (∆Head, ∆Tail, and ∆Head-Tail; Fig. 4d), and then performed co-IP after co-transfection with Myc-NEURL3. The results revealed that the ∆Head mutant lost the ability to interact with NEURL3, suggesting that Vimentin interacts with NEURL3 through its head domain (Fig. 4e).

**Fig. 5:**
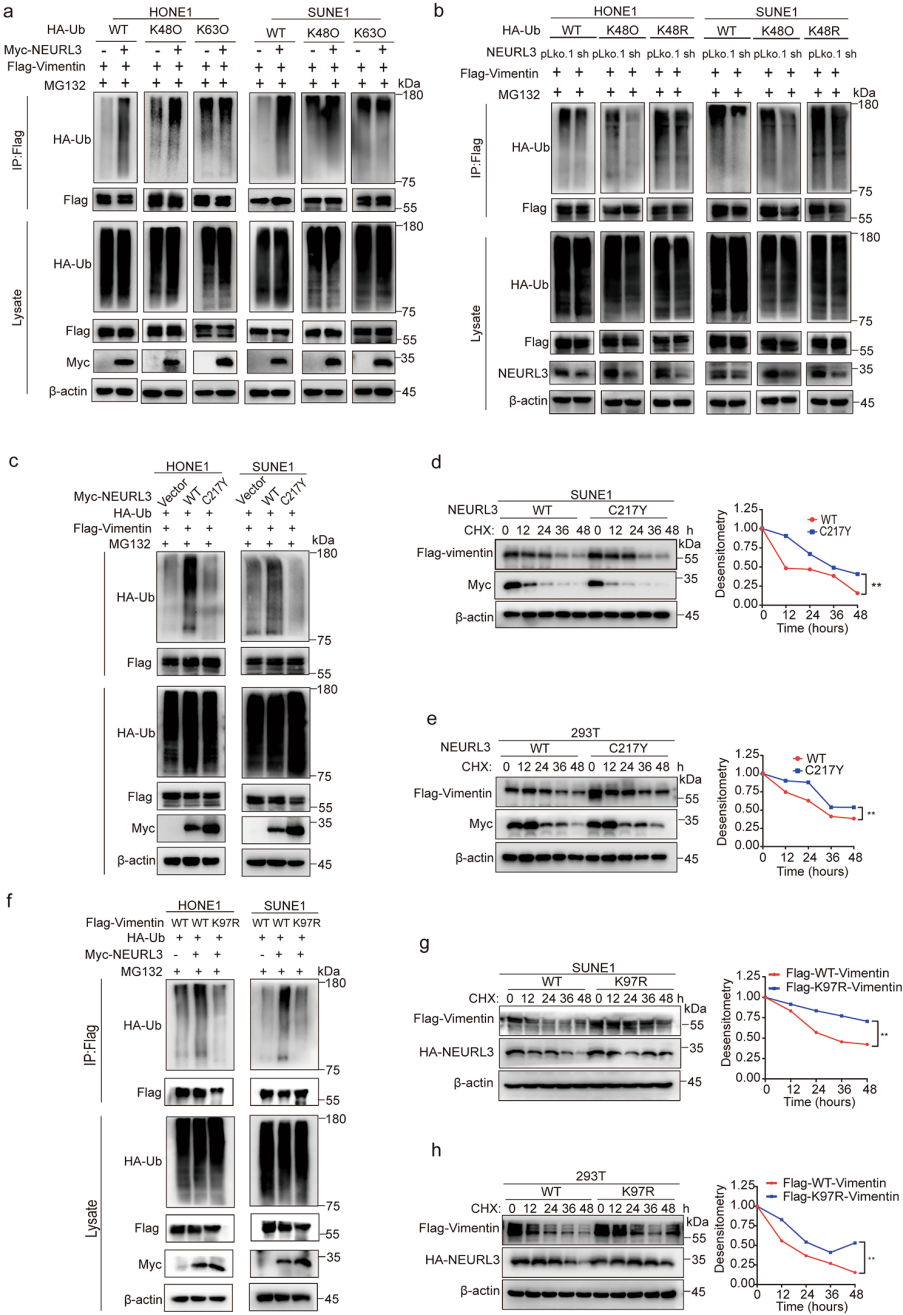
NEURL3 promotes K48-linked ubiquitination and degradation of Vimentin. **(a)** The ubiquitination level of exogenous Vimentin in HONE1 and SUNE1 cells co-transfected with Myc-NEURL3 or its empty vector, together with Flag-Vimentin plasmid and a vector encoding HA-WT-Ub or its mutants (HA-K48O-Ub or HA-K63O-Ub). **(b)** The ubiquitination level of exogenous Vimentin in HONE1 and SUNE1 cells co-transfected with shNEURL3 plasmid or its control vector, together with Flag-Vimentin plasmid and a vector encoding HA-WT-Ub or its mutants (HA-K48O-Ub or HA-K48R-Ub). **(c)** The ubiquitination level of exogenous Vimentin in HONE1 and SUNE1 cells co-transfected with empty vector, wild-type (WT) Myc-NEURL3 or its C217Y mutant, together with Flag-Vimentin and HA-WT-Ub. **(d-e)** Representative images and greyscale analyses of Vimentin protein levels after CHX treatment in SUNE1 **(d)** and 293T **(e)** cells co-transfected with WT Myc-NEURL3 or its C217Y mutant, together with Flag-Vimentin. **(f)** The ubiquitination level of exogenous Vimentin in HONE1 and SUNE1 cells co-transfected with Myc-NEURL3 or its empty vector, together with WT Flag-Vimentin or its K97R mutant and HA-WT-Ub. **(g-h)** Representative images and greyscale analyses of Vimentin protein levels after CHX treatment in SUNE1 **(g)** and 293T **(h)** cells co-transfected with Myc-NEURL3, together with WT Flag-Vimentin or its K97R mutant. Data of **d**, **e**, **g**, and **h** are shown as mean ± SD, and the *p*-values were determined by Student’s *t*-test (ns, no significance; *p < 0.05)

Then, we investigated the regulation of NEURL3 on Vimentin expression, and found that overexpression of NEURL3 decreased Vimentin protein levels while knockdown of NEURL3 increased Vimentin protein levels in NPC cells (Fig. 4f-h), but modulation of the NEURL3 expression had no effect on NEURL3 mRNA levels (Supplementary Fig. 4b-c). In addition, we revealed that overexpression of NEURL3 obviously increased E-cadherin expression while knockdown of NEURL3 led to a decrease in E-cadherin expression (Supplementary Fig. 5), demonstrating that NEURL3 could regulate the EMT process of NPC cells. We further found that overexpression of NEURL3 significantly facilitated Vimentin protein degradation while knockdown of NEURL3 remarkably suppressed Vimentin protein degradation in NPC cells after the cycloheximide (CHX) treatment (Fig. 4i-j). Since that the Ubiquitin-proteasome and autophagy-lysosome pathways are two main intracellular protein degradation manners within the eukaryotic cells, we treated NPC cells with the proteasome inhibitor MG132 or lysosome inhibitor chloroquine (CQ) after transfecting with HA-NEURL3 plasmid or empty vector. The results demonstrated that NEURL3-mediated protein degradation of Vimentin was reversed by MG132, but not by CQ (Fig. 4k-l), indicating that NEURL3 promotes the protein degradation of Vimentin through the ubiquitin-proteasome pathway.

### NEURL3 promotes K48-linked ubiquitination and degradation of vimentin

We then assessed the impact of NEURL3 on the ubiquitination of Vimentin by denatured immunoprecipitation (IP), and observed that overexpression of NEURL3 obviously increased the polyubiquitination of Flag-tagged Vimentin protein (Fig. 5a). Furthermore, overexpression of NEURL3 specifically promoted the K48-linked polyubiquitination but not the K63-linked polyubiquitination of Vimentin in HONE and SUNE1 cells (Fig. 5a). Conversely, knockdown of NEURL3 reduced the K48-linked polyubiquitination of vimentin (Fig. 5b). Subsequently, we obtained two ubiquitin (Ub) mutant plasmids: HA-Ub-K48O and HA-Ub-K48R, and found that knockdown of NEURL3 enhanced the ubiquitination of Vimentin when NPC cells were co-transfected with HA-Ub-K48O plasmid, but not the HA-Ub-K48R (Fig. 5b), demonstrating that NEURL3 promotes K48-linked ubiquitination of Vimentin.

Considering the well-established role of the cysteine-rich motif as the active site for E3 ligase activity, we constructed an enzyme inactive plasmid of NEURL3 with a mutant (C217Y) by substituting the cysteine residue with tyrosine as previously reported [27]. Ubiquitin assay revealed that NEURL3 (C217Y) lost its ability to ubiquitinate Vimentin (Fig. 5c), indicating that NEURL3 promotes the polyubiquitination of NEURL3 depending on its E3 ligase activity. Consistent with these findings, NEURL3 (C217Y) greatly prolonged the half-life of Vimentin compared with that of NEURL3 (WT) after CHX treatment (Fig. 5d-e).

NEURL3 specifically bound to the head domain of Vimentin but not to the rod and tail domains (Fig. 4e). Since ubiquitination modification primarily occurs at lysine residues within target proteins, we identified a lysine residue (K87) in the head domain of Vimentin, which is previously reported to be ubiquitinated by other E3 ligases [33, 34]. We generated a Vimentin plasmid with a mutant (K97R) by substituting the lysine residue with alanine, and observed that overexpression of NEURL3 had no impact on the polyubiquitination of Vimentin (K97R) (Fig. 5f). Moreover, overexpression of NEURL3 enhanced the degradation of Vimentin (WT), but not Vimentin (K97R) when NPC and 293T cells were treated with CHX (Fig. 5g-h). These findings indicate that NEURL3 ubiquitinates Vimentin at K97 in a manner dependent on its E3 ligase activity and thus promotes the protein degradation of Vimentin.

### Restoration of vimentin reverses the tumor suppressive effect of NRURL3

**Fig. 6:**
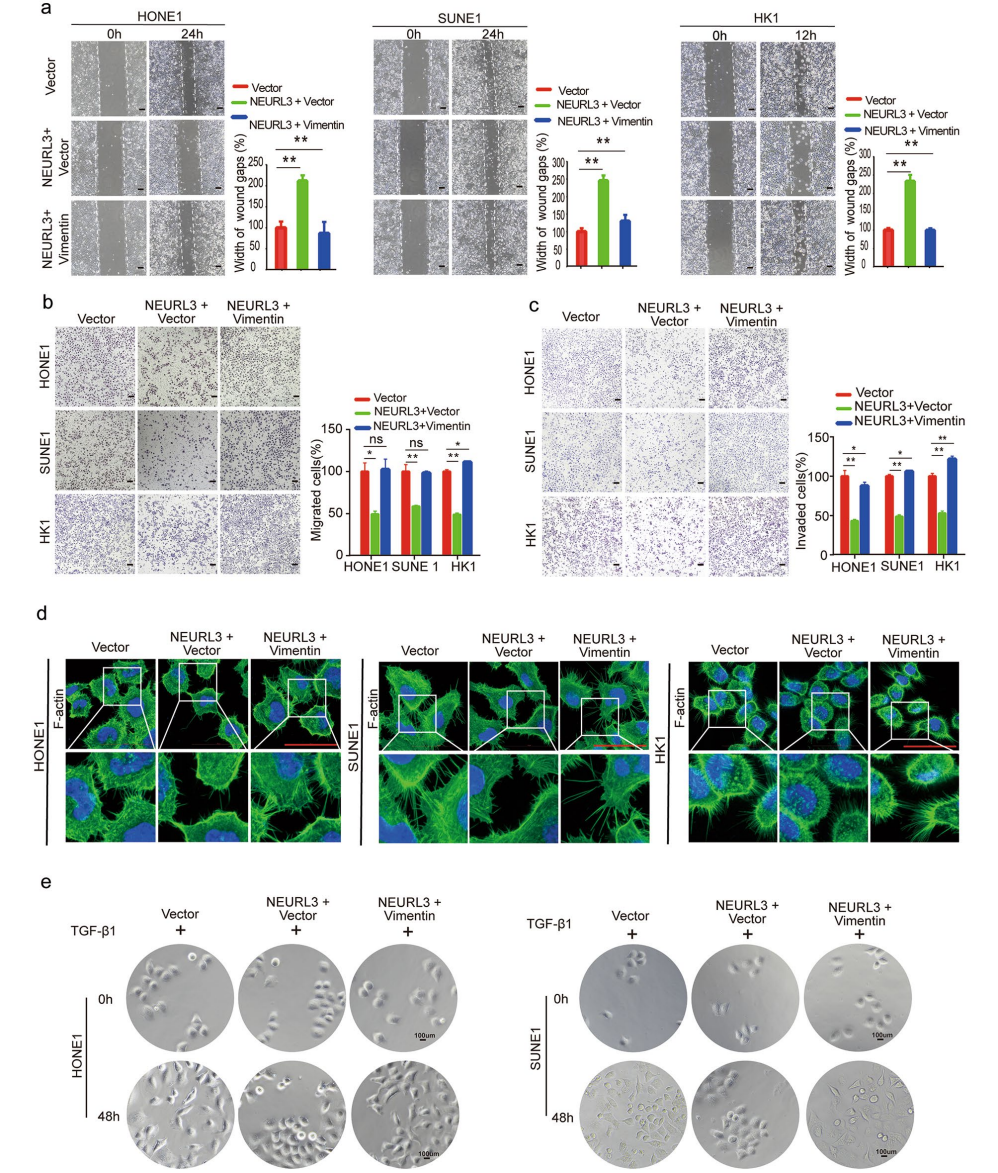
Restoration of Vimentin reverses the tumor suppressive effect of NRURL3. HONE1, SUNE1, and HK1 cells were transiently co-transfected with HA-NEURL3 or empty vector together with Flag-Vimentin or empty vector. **(a)** The migratory abilities of transfected NPC cells were determined by wound healing assay. **(b-c)** The migratory **(b)** and invasive **(c)** abilities of transfected NPC cells were determined by Transwell assay. **(d)** IF staining showing the pseudopods on transfected NPC cells. **(e)** The morphology changes induced by TGF-β1 in transfected NPC cells. Scale bar, 50 μm. Data of **a**, **b**, and **c** are shown as mean ± SD, and the *p*-values were determined by Student’s *t*-test (*p < 0.05)

To validate whether NEURL3 inhibits NPC cell migration, invasion, and EMT through promoting Vimentin degradation, we co-transfected NPC cells with NEURL3-overexpressing, Vimentin-overexpressing, or empty vector plasmids (Supplementary Fig. 6a), and then did in vitro functional experiments. The results of the wound healing and Transwell assays showed that overexpression of Vimentin could completely rescue the suppressive effects of NEURL3 overexpression on NPC cell migration and invasion (Fig. 6a-c). Additionally, the length of cellular pseudopods and TGF-β1-induced EMT phenotype of NPC cells that were suppressed after NEURL3 overexpression were fully reversed by overexpression of Vimentin (Fig. 6d-e; Supplementary Fig. 6b). These findings show that overexpression of Vimentin can reverse the suppressive effect of NEURL3 on metastasis and EMT in NPC.

**Fig. 7:**
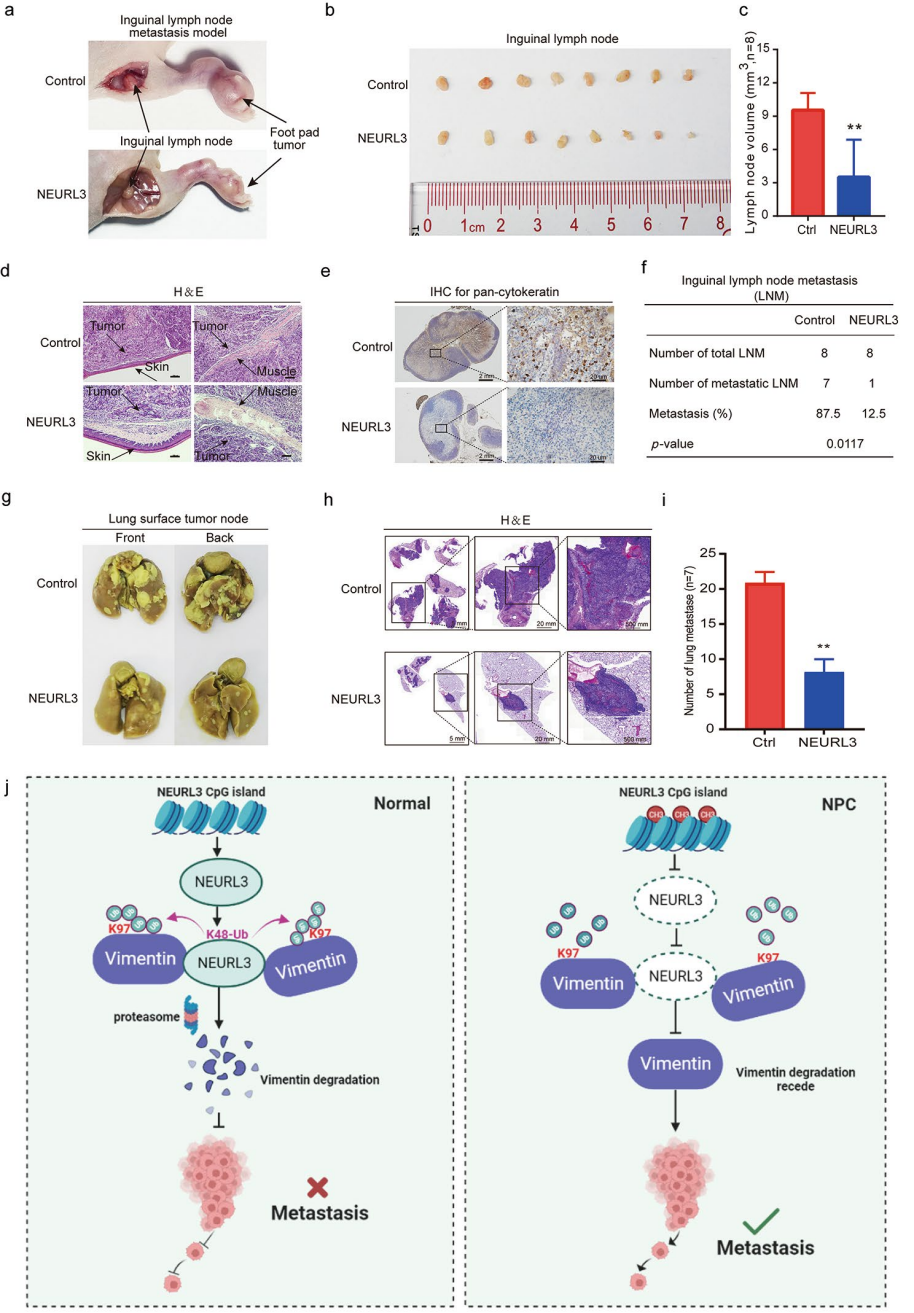
NEURL3 inhibits NPC metastasis in vivo. SUNE1 cells stably expressing HA-NEURL3 or its empty vector were subcutaneously injected into foot pads of mice to establish an inguinal lymph node metastasis model. **(a)** Representative images of the formed footpad tumors and inguinal lymph nodes. **(b-c)** Representative images of inguinal lymph nodes **(b)**, and statistical analysis of the volume of lymph nodes **(c)** between the two groups. Data is shown as mean ± SD, and the *p*-value was determined by Student’s *t*-test (*p < 0.05). **(d)** H&E staining showing the infiltration of tumor cells into skin and muscle of footpad tumors (Scale bar, 100 μm). **(e)** The infiltration of cancer cells into inguinal lymph nodes as determined by a positive of pan-cytokeratin IHC staining (Scale bar, 2 mm or 20 μm). **(f)** Comparison of the positive ratios of the inguinal lymph node metastasis between the two groups. *P*-value was determined by Chi-square test (*p < 0.05). SUNE1 cells stably expressing HA-NEURL3 or its empty vector were inoculating into tail veins of mice to establish a lung metastatic colonization model. **(g)** Representative images of macroscopic metastatic nodules formed on the lung surfaces of mice in the two groups. **(h-i)** Representative images (**h**; Scale bars, 5 mm, 2 mm, and 20 μm) and statistical analysis **(i)** of metastatic nodules formed in the lungs of mice determined by H&E staining. Data is shown as mean ± SD, and the *p*-value was determined by Student’s *t*-test (*p < 0.05). **(j)** Proposed working model. NEURL3 promotes the degradation of Vimentin protein in a ubiquitin-proteasome pathway, inhibiting NPC metastasis (left). In NPC, promoter hypermethylation of NEURL3 causes its downregulation, enabling increased expression of Vimentin to promote NPC metastasis (right)

### NEURL3 inhibits NPC metastasis in vivo

To investigate the role of NEURL3 on NPC metastasis in vivo, we established an inguinal lymph node metastasis model by transplanting SUNE1 cells stably overexpressing NEURL3 or its control into the foot pads of nude mice (*n* = 8 in each group). The results showed that the volumes of the inguinal lymph nodes in the NEURL3-overexpressing group were smaller compared to those in the control group ((Fig. 7a-c; Supplementary Fig. 7a). H&E staining of foot pad tumors indicated that the NEURL3 group exhibited less aggressiveness, as showed by a fewer invasion towards the skin or muscle, in comparison to the control group (Fig. 7d; Supplementary Fig. 7b). Furthermore, IHC staining showed a less ratio of inguinal lymph nodes metastasis, as evidenced by a less of positive of pan-cytokeratin, in the NEURL3-overexpression group (Fig. 7e-f; Supplementary Fig. 7c).

In addition, we constructed a lung metastatic colonization model by inoculating SUNE1 cells stably overexpressing NEURL3 or its empty control into the tail veins of nude mice (*n* = 8 in each group). The results showed that overexpression of NEURL3 resulted in a remarkable reduction of metastatic nodules formed on the surface of lungs (Fig. 7g; Supplementary Fig. 7d). In Addition, H&E staining confirmed that mice in the NEURL3-overexpressing group had fewer and smaller metastatic nodules in their lungs (Fig. 7h-i). Collectively, these findings demonstrate that NEURL3 inhibits NPC metastasis in vivo.

## Discussion

In our present study, we demonstrate that the promoter region of NEURL3 was generally hypermethylated in NPC, leading to a decreased expression of NEURL3. Low expression of NEURL3 was associated with poor prognosis and indicated tumor metastasis in NPC patients. Overexpression of NEURL3 significantly inhibited NPC metastasis and EMT in vitro and in vivo. Mechanistically, NEURL3 facilitated the K48-linked polyubiquitination of Vimentin at the K97 site dependent on its E3 ligase activity, and thus promoted the protein degradation of Vimentin. Restoration of the Vimentin expression could fully rescue the suppressive effect of NEURL3 overexpression on NPC cell migration and invasion. Our study uncovers a novel mechanism by which NEURL3 promotes Vimentin degradation to inhibit NPC metastasis (Fig. 7j), thereby providing a promising therapeutic target for NPC treatment.

NEURL3, also known as lung-inducible neuralized-related C3HC4 RING domain protein (LINCR), is a 262-amino-acid E3 ubiquitin ligase that contains a neuralized homology repeat (NHR) domain, a RING finger structural domain, and a C-terminus [25, 27, 28]. NEURL3 is initially identified and characterized in lipopolysaccharide-induced mouse alveolar epithelial cells [25, 26]. Recent research has unveiled the ability of NEURL3 to augment host antiviral response by catalyzing K63-linked ubiquitination of interferon regulatory factor 7 (IRF7) [27]. Additionally, NEURL3 is shown to function as an antiviral effector against the assembly of hepatitis C virus [28]. Moreover, NEURL3 assumes a critical function in governing pulmonary embryogenesis and spermatogenesis [29, 30]. However, the biological function of NEURL3 in the tumorigenesis and progression has not been thoroughly investigated. Here, based on two genome-wide methylation microarray datasets, we demonstrated that the promoter region of NEURL3 was hypermethylated in NPC and resulted in its downregulation. Furthermore, we unequivocally revealed an inhibitory impact of NEURL3 on NPC metastasis, broadening our understanding of the role of NEURL3 in tumor biology.

In terms of mechanism, a study reveals that NEURL3 binds to the envelope glycoprotein E1, leading to the disruption of E1/E2 heterodimerization and resulting in the suppression of assembly of hepatitis C virus [28]. Notably, the NHR domain of NEURL3 is crucial for this inhibitory effect, while the RING domain is not involved [28]. Interestingly, the RING domain is widely recognized as a characteristic of the E3 ubiquitin ligases, dictating their activity [34]. This may suggest that NEURL3 functions as a putative E3 ligase, but its specific substrates have not been clearly identified. A study reports that the C217Y mutation in the RING domain of NEURL3 can inactivate its ligase function, thus decreasing the IRF7-mediated immune response [27]. In this study, we found that mutating the RING domain C217Y site of NEURL3 diminished the ubiquitination level of Vimentin in NPC cells. This site served as the active site of ligase for NEURL3-mediated ubiquitination, leading to the protein degradation of Vimentin. K48-linked and K63-linked polyubiquitin chains are the main types of ubiquitin linkages [35]. It has been reported that NEURL3 could promote the K63-linked polyubiquitination of IRF7 at its lysine 375 [27]. In our present study, we revealed that NEURL3 specifically induced the K48-linked polyubiquitination of Vimentin, rather than the K63-linked polyubiquitination. Our findings underscore the wide-ranging functional capabilities of NEURL3 as an E3 ubiquitin ligase in governing the degradation of target proteins, and offer a partial explanation for the anti-tumor biological role of NEURL3 in NPC.

Vimentin is a well-established marker of EMT and it serves as a crucial component for the initiation of cancer invasion and metastasis cascades. Elevated level of Vimentin has been detected in aggressive epithelial cancers, and is closely associated with an increased likelihood of metastasis and poor prognosis across various types of cancers, including NPC [36–38]. It has been reported that polyubiquitination may be a crucial post-translational modification that involved in regulating Vimentin degradation. In breast cancer cells, RING finger protein 208 (RNF208) is found to play an inhibitory role in metastasis by interacting with phosphorylated Vimentin Ser39 residues. Specifically, RNF208 targets the lysine 97 residues within the head domain of Vimentin for its ubiquitination modification, resulting in proteasomal degradation of Vimentin [33]. Another research reports the impact of FERM domain-containing protein 3 (FRMD3) on vimentin degradation, resulting in anti-proliferative and anti-metastatic roles in breast cancer [34]. The N-terminal ubiquitin-like domain of FRMD3 interacts with the head domain of Vimentin and binds to ubiquitin-protein ligase E3A (UBE3A), ultimately promoting polyubiquitination-mediated proteasomal degradation of Vimentin [34]. However, no studies have reported the mechanism of ubiquitination-mediated Vimentin protein degradation in NPC. Our study demonstrated that the RING domain of NEURL3 mediated Vimentin ubiquitination degradation by targeting the lysine 97 site within the head domain of Vimentin. This targeting of lysine 97 by NEURL3 for Vimentin degradation aligns with the reported action sites of other E3 ubiquitin ligases involved in Vimentin degradation.

Metastasis is a major obstacle in the treatment of NPC and remains the main cause of cancer related mortality. Vimentin is at the heart of EMT-mediated metastasis [39], and it can affect the expression of genes for EMT inducers, such as Snail, Slug, Twist, and ZEB1/2, as well as key epigenetic factors [39]. Moreover, it has demonstrated the capacity of Vimentin to induce the expression of self-renewability related genes for suppressing cellular differentiation and enhancing pluripotent potential [40]. The increased stemness of the cancer stem cells can facilitate tumor spread and render resistant to anti-tumor treatments [41]. In addition, various missense and frameshift mutations identified in Vimentin in human cancers may contribute to the metastatic spread of tumor cells [40]. Here, we demonstrated that NEURL3 promoted the ubiquitination and degradation of Vimentin to suppress NPC invasion and metastasis. We thus propose that targeting Vimentin representing a promising therapeutic approach to limit cancer growth and spread, resulting in reduced mortality of NPC.

Indeed, Vimentin serves as a pro-oncogenic factor that negatively impacts the prognosis of various tumor types. Beyond its involvement in tumor cell EMT and metastasis, Vimentin plays a critical role in tumor angiogenesis, immune infiltration, and immune suppression [42]. Notably, it has been found that a correlation between Vimentin and PD-L1 expression exists in tumor tissues of non-small cell lung carcinoma, and the co-positivity for the two markers is associated with a discernible inclination toward poorer patient prognosis [43]. Consequently, we hypothesis that targeted down-regulation of Vimentin expression can not only inhibit NPC metastasis but also potentially enhance the effectiveness of immunotherapy. Immunotherapy holds significant promise in enhancing the clinical effectiveness of NPC treatment, and it is a prominent area of research in the field [44, 45]. As mentioned above, evidence suggests that NEURL3 plays a crucial role in innate immunoregulation. For instance, NEURL3 boosts the host’s antiviral response by facilitating K63-linked ubiquitination of IRF7 [27]. Therefore, further investigation into the involvement of NEURL3 in the immunoregulation of NPC would shed light on its clinical significance in modulating NPC immunity.

Undoubtedly, this article has its shortcomings. Firstly, our exploration of the mechanism behind NEURL3’s inhibition of NPC EMT and metastasis solely focused on the regulation of Vimentin by NEURL3, without delving into potential intermediate molecules. Secondly, due to time, effort, and resource constraints, we did not develop specific methods that up-regulate NEURL3, thus potentially limiting its clinical applicability. These limitations highlight future research directions that we intend to pursue.

## Conclusions

In conclusion, we demonstrate that NEURL3 is downregulated in NPC because of its promoter hypermethylation, leading to a poor prognosis and posttreatment distant metastasis in NPC patients. NEURL3 recruits and promotes the protein degradation of Vimentin through the ubiquitin-proteasome pathway, thus suppressing NPC metastasis by inactivating EMT. Our study reveals a novel mechanism by which NEURL3 inhibits NPC metastasis and provides novel therapeutic targets for NPC patients. Drawing upon our findings and existing researches, modulating E3 ubiquitin ligase or deubiquitinases to inhibit EMT and metastasis in NPC holds immense promise for enhancing the clinical effectiveness of NPC treatment.

### Electronic supplementary material

Below is the link to the electronic supplementary material.

Supplementary Material 1

Supplementary Material 2

Supplementary Material 3

## Data Availability

The data and materials of this research are available from the corresponding author on reasonable request. The key raw data have been deposited to the Research Data Deposit public platform (http://www.researchdata.org.cn/), with an approval number of RDDB2023949110.
