# Supplementary material for: The E3 ligase NEURL3 suppresses epithelial-mesenchymal transition and metastasis in nasopharyngeal carcinoma by promoting vimentin degradation
Source: J Exp Clin Cancer Res. 2024 Jan 9;43:14. doi: 10.1186/s13046-024-02945-9 (PMC10775674; doi:10.1186/s13046-024-02945-9)
Supplement: Supplementary file 3 — Supplementary Material 3 [file 13046_2024_2945_MOESM3_ESM.docx]

**Supplementary Information**

**The E3 ligase NEURL3 suppresses epithelial-mesenchymal transition and metastasis in nasopharyngeal carcinoma by promoting Vimentin degradation**

**Zhou et al.**


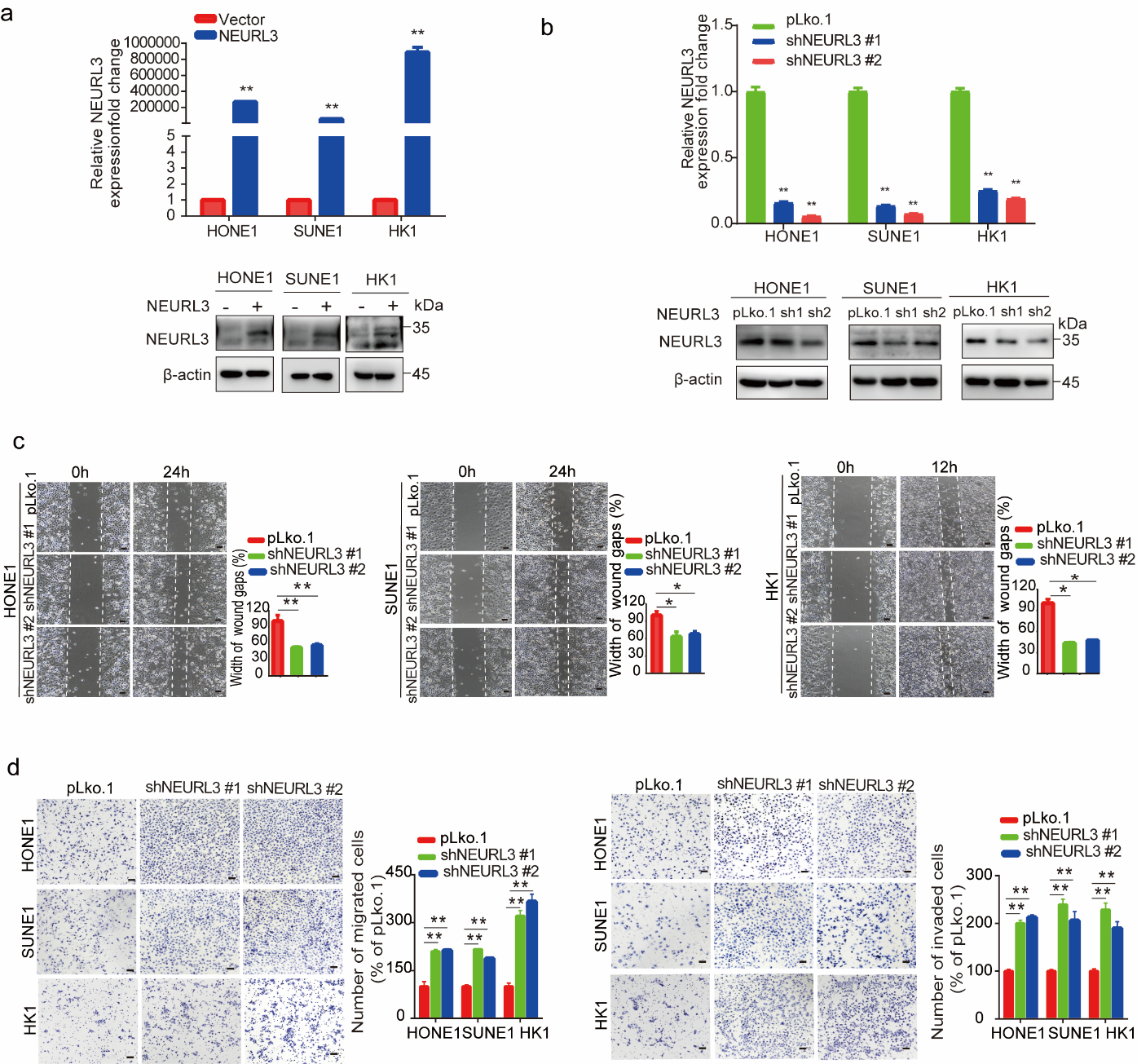


**Supplementary Fig. 1: Knockdown of NEURL3 promotes NPC cell migration and invasion**

**a.** Relative mRNA and protein levels of NEURL3 in HONE1, SUNE1, and HK1 cells transfected with HA-NEURL3 plasmid or its empty vector. **b.** Relative mRNA and protein levels of NEURL3 in HONE1, SUNE1, and HK1 cells transfected with shNEURL3 plasmids or its control vector. **c.** The migratory abilities of HONE1, SUNE1, and HK1 cells transfected with shNEURL3 plasmids or its control vector determined by wound healing assay. **d.** The migratory and invasive abilities of HONE1, SUNE1, and HK1 cells transfected with shNEURL3 plasmids or its control vector determined by Transwell assay. Data are shown as mean ± SD, and the *p*-values were determined by Student’s t-test (**p* < 0.05).


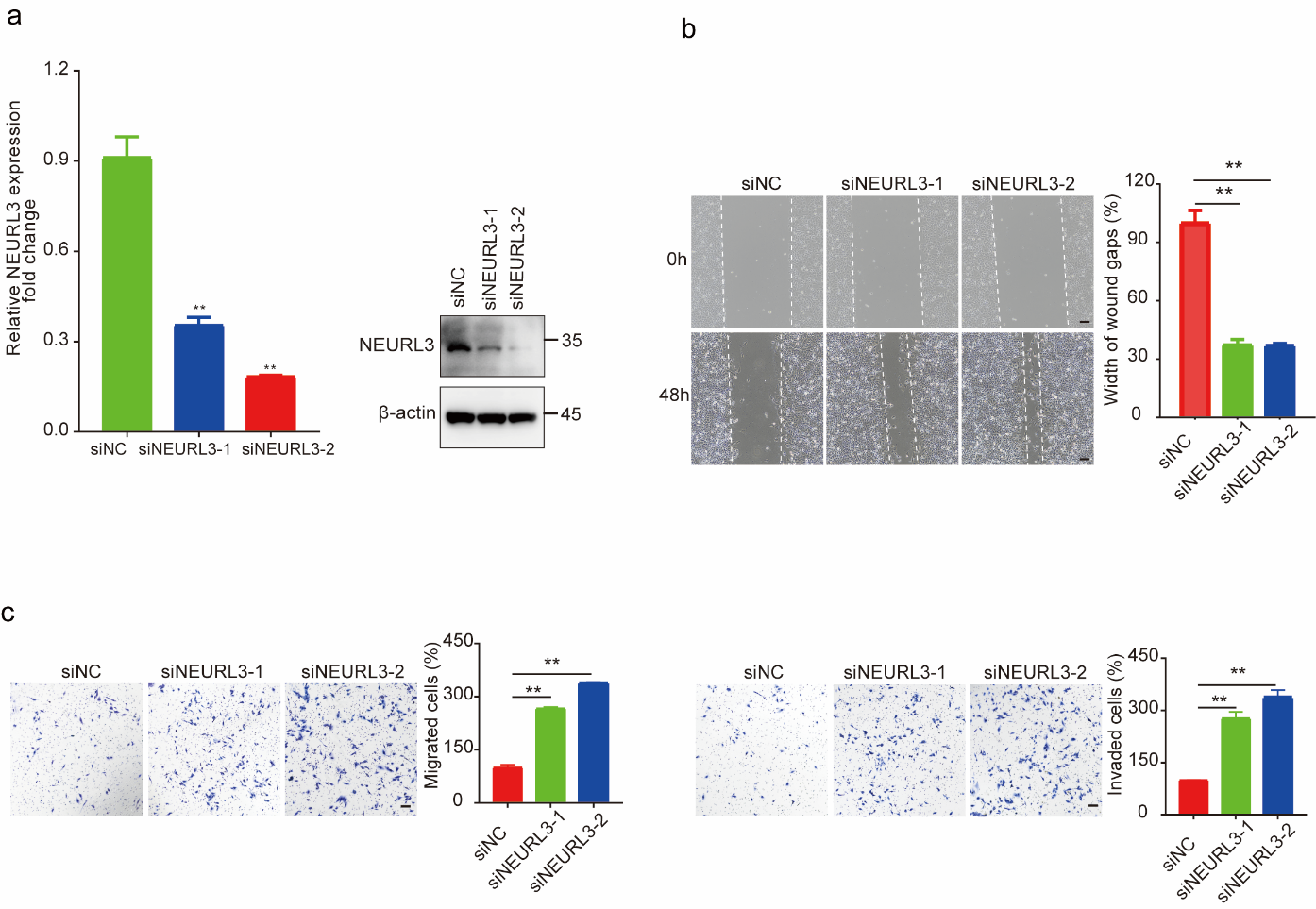


**Supplementary Fig. 2: Knockdown of NEURL3 promotes NP69 cell migration and invasion**

**a.** Relative mRNA and protein levels of NEURL3 in NP69 cells transfected with siNEURL3 or its scramble control. **b.** The migratory abilities of NP69 cells transfected with siNEURL3 or its scramble control determined by wound healing assay. **c.** The migratory and invasive abilities of NP69 cells transfected with siNEURL3 or its scramble control determined by Transwell assay. Data are shown as mean ± SD, and the *p*-values were determined by Student’s t-test (**p* < 0.05).


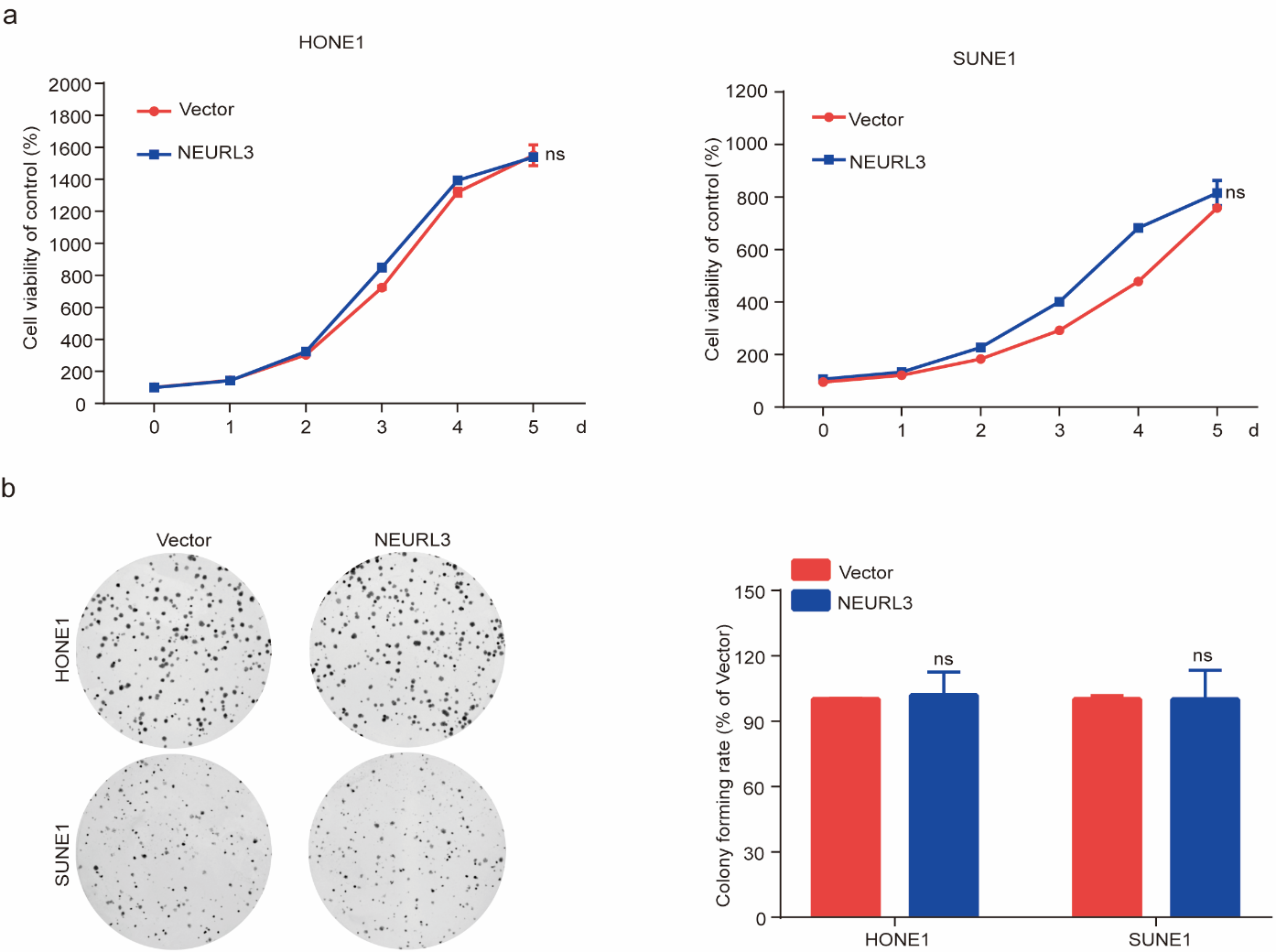


**Supplementary Fig. 3: Overexpression of NEURL3 has no effect on NPC cell proliferation**

**a.** The cell variabilities of HONE1 and SUNE1 cells transfected with HA-NEURL3 plasmid or its empty vector determined by CCK-8 assay. **b.** Representative images (left) and quantification analysis (right) of the colonies formed in HONE1 and SUNE1 cells transfected with HA-NEURL3 plasmid or its empty vector, determined by colony formation assay. The data are shown as mean ± SD, and the *p*-values were determined by Student’s t-test (ns, no significance; **p* < 0.05).


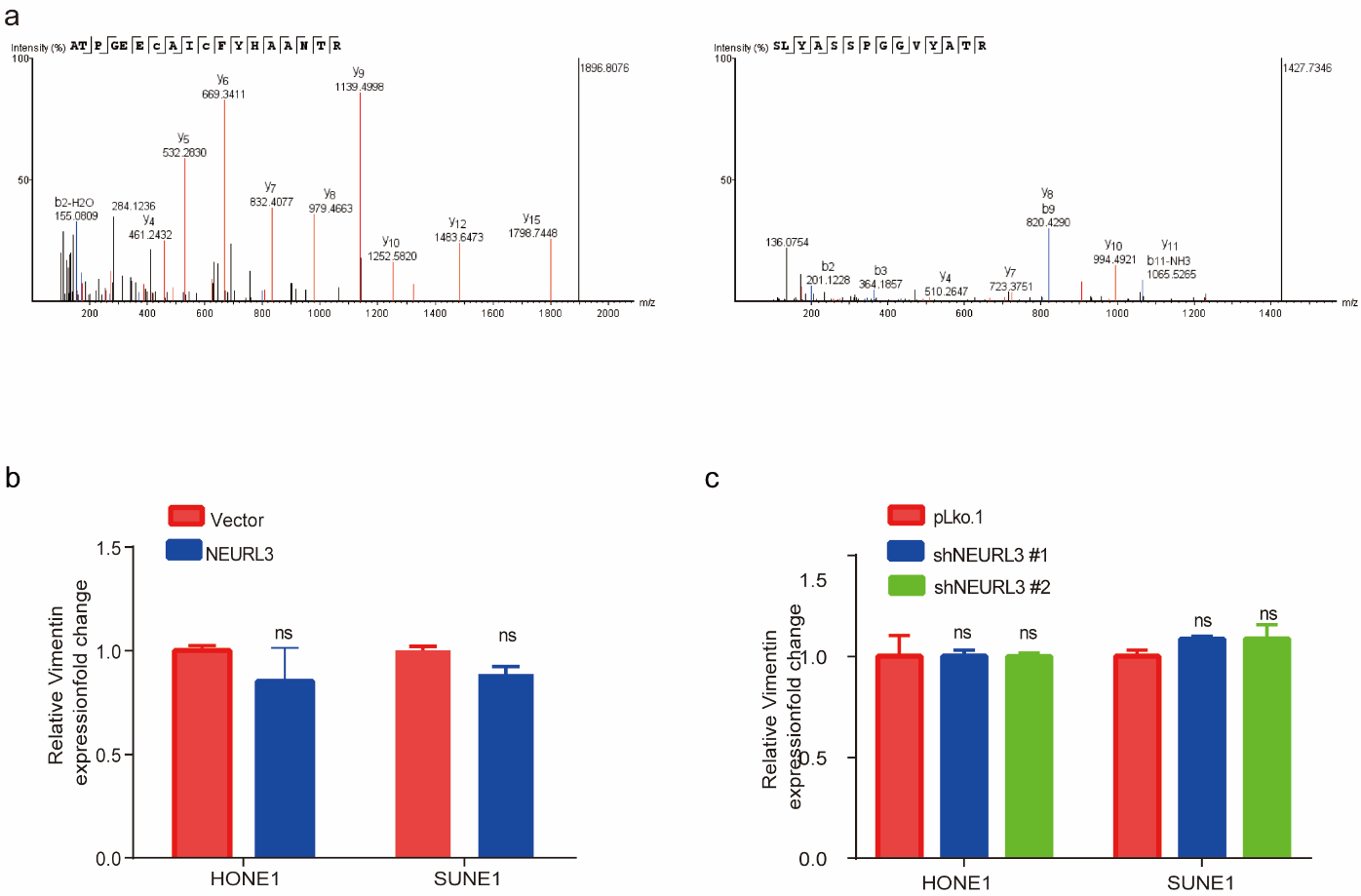


**Supplementary Fig. 4: The NEURL3 junction-specific peptides and the Vimentin mRNA level in NPC cells transfected with indicated plasmids.**

**a.** NEURL3 (left) junction-specific peptide of Vimentin (right) was identified with immunoprecipitation of anti-HA antibody in SUNE1 cells transiently transfected with HA-NEURL3. **b.** The relative mRNA level of Vimentin in HONE1 and SUNE1 cells transfected with HA-NEURL3 plasmid or its empty vector. **c.** The relative mRNA level of Vimentin in HONE1 and SUNE1 cells transfected with shNEURL3 plasmids or its vector control. The data are shown as mean ± SD, and the *p*-values were determined by Student’s t-test (ns, no significance).


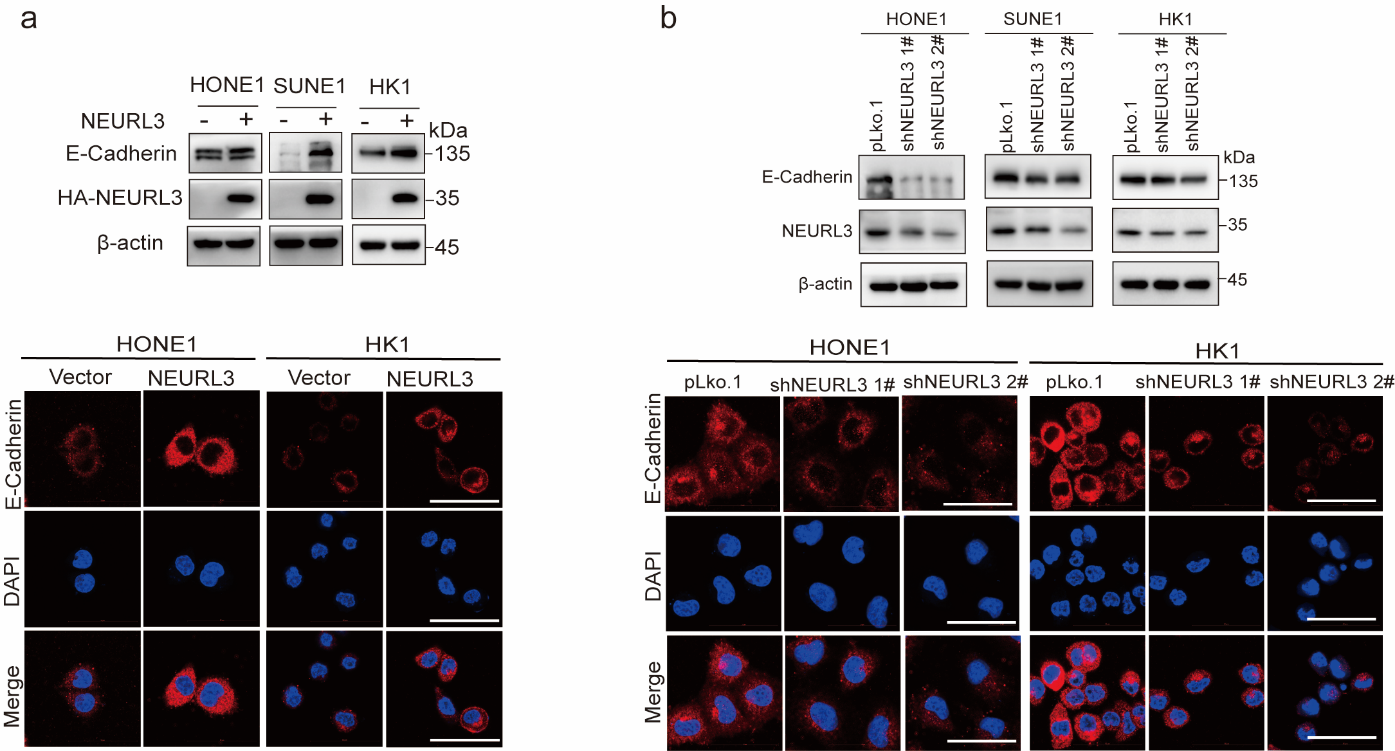


**Supplementary Fig. 5: E-Cadherin expression in NPC cells after modulation NEURL3 expression.**

**a.** Western blot (upper) and immunofluorescence (lower) assays were performed to assess the expression levels of E-cadherin in HONE1 and HK1 cells transfected with HA-NEURL3 plasmid or its empty vector. **b.** Western blot (upper) and immunofluorescence (lower) assays were performed to assess the expression levels of E-cadherin in HONE1 and HK1 cells transfected with shNEURL3 plasmids or its control vector.


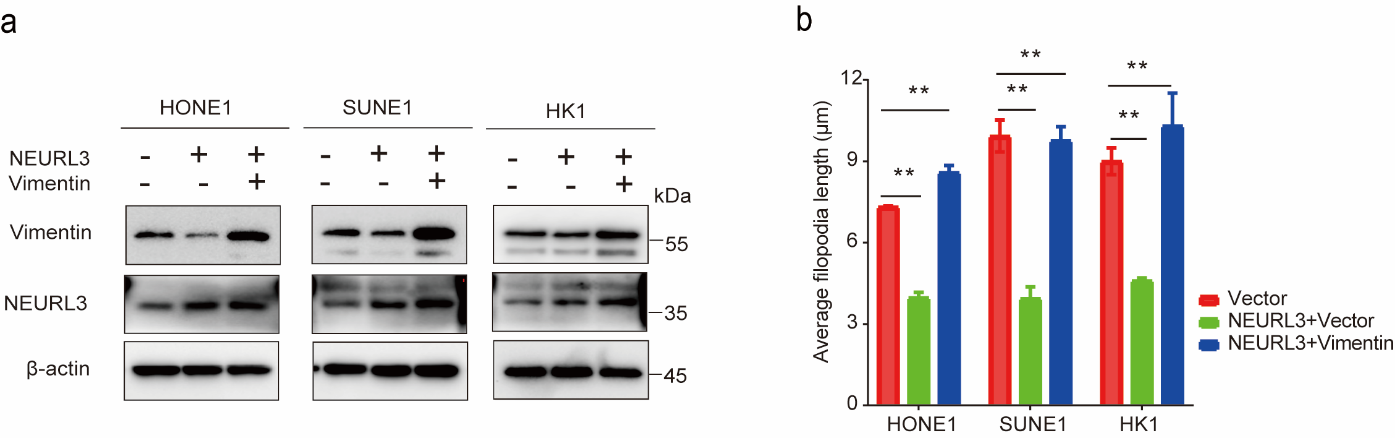


**Supplementary Fig. 6:** **Vimentin protein levels (a) and filopodia length (b) in HONE1, SUNE1, and HK1 cells transfected with HA-NEURL3 or its empty vector, in conjunction with Flag-Vimentin or its empty vector.**


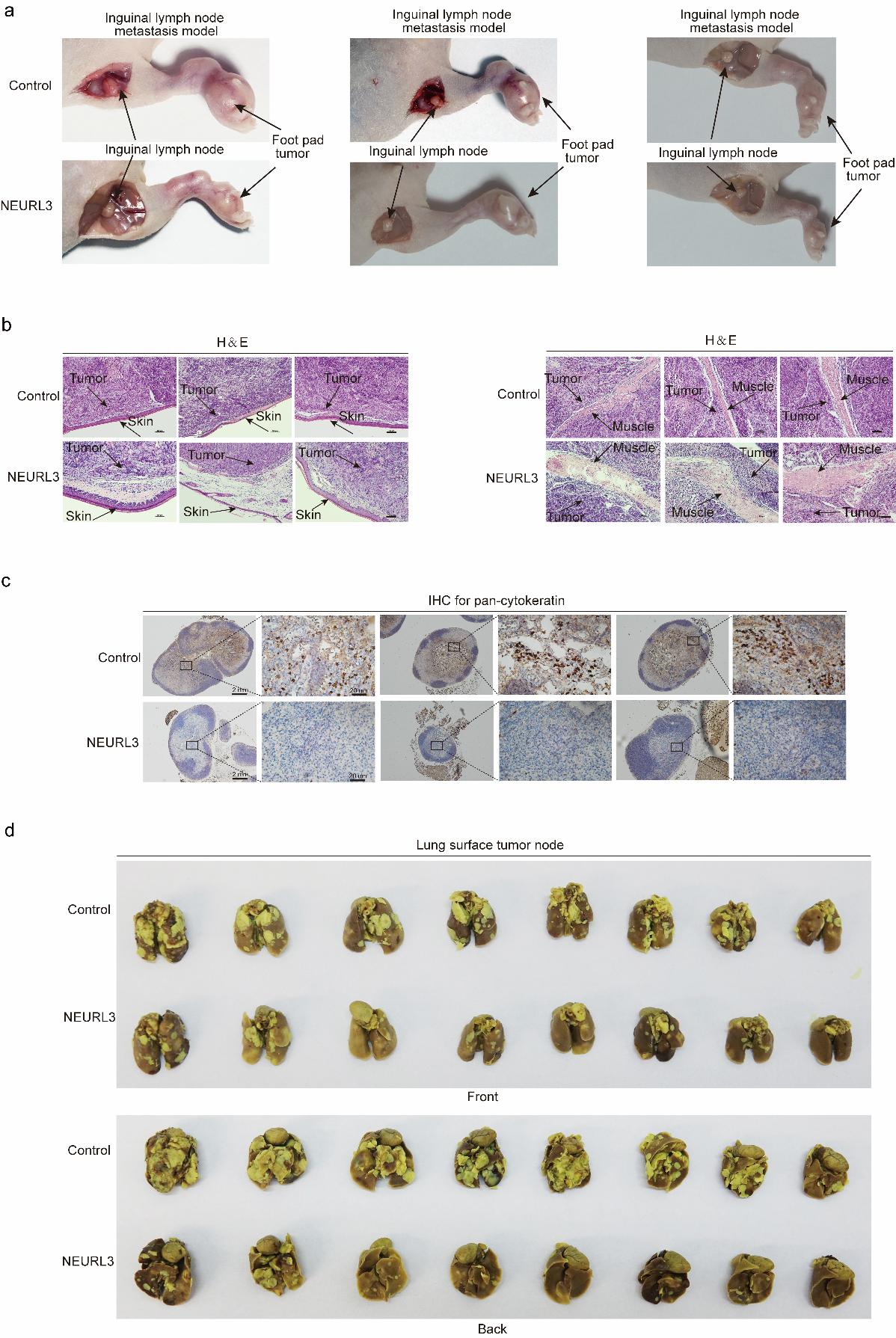


**Supplementary Fig. 7: NEURL3 inhibits NPC metastasis *in vivo***

**a.** Representative images of the formed footpad tumors and inguinal lymph nodes. **b.** H&E staining showing the infiltration of tumor cells into skin and muscle of footpad tumors (Scale bar, 100 μm). **c.** The infiltration of cancer cells into inguinal lymph nodes as determined by a positive of pan-cytokeratin IHC staining (Scale bar, 2 mm or 20 μm). **g.** Representative images of macroscopic metastatic nodules formed on the lung surfaces of mice in the two groups.

**Supplementary Table 1: Relationship between NEURL3 expression and clinical characteristics of NPC patients (N = 212)**

| **Variable** | **NEURL3** | ***p*-value** |  |
| --- | --- | --- | --- |
|  | **Low expression**  **(N, %)** | **High expression (N, %)** |  |
| **Age** |  |  |  |
| ≤ 45 years | 58 (54.7) | 51 (48.1) | 0.410 |
| > 45 years | 48 (45.3) | 55 (51.9) |  |
| **Gender** |  |  |  |
| Male | 82 (77.4) | 77 (72.6) | 0.526 |
| Female | 24 (22.6) | 29 (27.4) |  |
| **TNM Stage** |  |  |  |
| III | 72 (67.9) | 65 (61.3) | 0.389 |
| IV | 34 (32.1) | 41 (38.7) |  |
| WHO Type |  |  |  |
| 2 | 7 (6.6) | 1 (0.9) | 0.065 |
| 3 | 99 (93.4) | 105 (99.1) |  |
| **VCA IgA** |  |  |  |
| < 1:80 | 11 (10.4) | 15 (14.2) | 0.531 |
| ≥ 1:80 | 95 (89.6) | 91 (85.8) |  |
| **EA IgA** |  |  |  |
| < 1:10 | 25 (23.6) | 22 (20.8) | 0.741 |
| ≥ 1:10 | 81 (76.4) | 84 (79.2) |  |
| **Relapse-free survival** |  |  |  |
| Live | 69 (65.1) | 86 (81.1) | **0.008** |
| Deaths | 37 (34.9) | 20 (18.9) |  |
| **Distant metastasis-free survival** |  |  |  |
| Live | 81 (76.4) | 97 (91.5) | **0.004** |
| Deaths | 25 (23.6) | 9 (8.5) |  |
| **Overall survival** |  |  |  |
| Live | 73 (68.9) | 90 (84.9) | **0.009** |
| Deaths | 33 (31.1) | 16 (15.1) |  |

Abbreviations: VCA IgA, viral capsid antigen immunoglobulin A; EA IgA, early antigen immunoglobulin A; HR, hazard ratio. Bold values indicate *p* < 0.05. The *p-*value was determined by χ^2^ or Fisher’s exact tests.

**Supplementary Table 2: Primers used in this study**

| **Name** | **Sequences (5'-3')** |
| --- | --- |
| **BSP PCR primers** | |
| NEURL3-F | GGGGAGGTTTTAGTTGTGTAGAGA |
| NEURL3-R | CCCCTCCCCACTTTAAAAACTC |
| **BSP sequencing primer** | |
| NEURL3 | GTTTTAGTTGTGTAGAGATTGA |
| **RT-qPCR primers** | |
| NEURL3-F | ATGGGACCTCAGCAACAAGGCT |
| NEURL3-R | AAGACCCGCCAGGCACAGTATC |
| Vimentin-F | TCTAGGAGGAGATGCGG |
| Vimentin-R | GGTCAAGACGTGCCAGAGAC |
| GAPDH-F | GTCTCCTCTGACTTCAACAGCG |
| GAPDH-R | ACCACCCTGTTGCTGTAGCCAA |
| **shRNA sequences** | |
| shNEURL3-1-F | CCGGTTATCTGCAAGGAATGTATAAACTCGAGTTTATACATTCCTTGCAGATATTTTTG |
| shNEURL3-1-R | AATTCAAAAATATCTGCAAGGAATGTATAAACTCGAGTTTATACATTCCTTGCAGATAA |
| shNEURL3-2-F | CCGGTATGCATCTGAAGATGATTTAACTCGAGTTAAATCATCTTCAGATGCATTTTTTG |
| shNEURL3-2-R | AATTCAAAAAATGCATCTGAAGATGATTTAACTCGAGTTAAATCATCTTCAGATGCATA |

**Supplementary Table 3: Specific interacting proteins of HA co-immunoprecipitation in HA-NEURL3 SUNE1 cells by LC−MS/MS**

| **Accession** | **-10lgP** | **Coverage (%) IP** | **#Peptides** | **#Unique** | **#Spec IP** | **Avg. Mass** | **Protein name** |
| --- | --- | --- | --- | --- | --- | --- | --- |
| **sp\|Q96EH8\|NEUL3_HUMAN** | **273.32** | **62** | **39** | **38** | **132** | **28789** | **NEURL3** |
| sp\|P60709\|ACTB_HUMAN | 236 | 41 | 25 | 8 | 55 | 41737 | ACTB |
| sp\|P63261\|ACTG_HUMAN | 236 | 41 | 25 | 8 | 55 | 41793 | ACTG1 |
| sp\|Q96C19\|EFHD2_HUMAN | 217.74 | 65 | 29 | 28 | 62 | 26697 | EFHD2 |
| sp\|Q04695\|K1C17_HUMAN | 210.93 | 36 | 19 | 7 | 24 | 48106 | KRT17 |
| sp\|P35908\|K22E_HUMAN | 210.46 | 38 | 25 | 17 | 35 | 65433 | KRT2 |
| sp\|P62701\|RS4X_HUMAN | 208.04 | 57 | 24 | 24 | 50 | 29598 | RPS4X |
| sp\|P13647\|K2C5_HUMAN | 207.75 | 36 | 26 | 7 | 37 | 62378 | KRT5 |
| sp\|P61247\|RS3A_HUMAN | 206.84 | 53 | 23 | 23 | 45 | 29945 | RPS3A |
| sp\|P47756\|CAPZB_HUMAN | 206.81 | 50 | 17 | 17 | 53 | 31350 | CAPZB |
| sp\|P02533\|K1C14_HUMAN | 204.41 | 37 | 20 | 2 | 27 | 51562 | KRT14 |
| **sp\|P08670\|VIME_HUMAN** | **203.37** | **51** | **25** | **18** | **34** | **53652** | **VIM** |
| sp\|Q15149\|PLEC_HUMAN | 202.32 | 6 | 32 | 30 | 38 | 531796 | PLEC |
| sp\|P18124\|RL7_HUMAN | 202.25 | 56 | 22 | 18 | 42 | 29226 | RPL7 |
| sp\|P62424\|RL7A_HUMAN | 201.7 | 47 | 18 | 15 | 29 | 29996 | RPL7A |
| sp\|P22087\|FBRL_HUMAN | 200.19 | 36 | 12 | 12 | 20 | 33784 | FBL |
| sp\|P15880\|RS2_HUMAN | 200.03 | 42 | 17 | 15 | 30 | 31324 | RPS2 |
| sp\|P07355\|ANXA2_HUMAN | 199.26 | 45 | 17 | 2 | 25 | 38604 | ANXA2 |
| sp\|Q96HS1\|PGAM5_HUMAN | 198.16 | 56 | 21 | 19 | 38 | 32004 | PGAM5 |
| sp\|P52907\|CAZA1_HUMAN | 194.1 | 38 | 15 | 8 | 46 | 32923 | CAPZA1 |
