# Supplementary material for: The E3 ligase NEURL3 suppresses epithelial-mesenchymal transition and metastasis in nasopharyngeal carcinoma by promoting vimentin degradation
Source: J Exp Clin Cancer Res. 2024 Jan 9;43:14. doi: 10.1186/s13046-024-02945-9 (PMC10775674; doi:10.1186/s13046-024-02945-9)
Supplement: Supplementary file 2 — Supplementary Material 2 [file 13046_2024_2945_MOESM2_ESM.doc]

Dear Editor and Reviewers,

Thank you very much for your important and supportive comments, questions and suggestions, which have greatly helped us to improve our study. Enclosed is the revised version of manuscript (Ref.: JECC-D-23-02306) entitled, “**The E3 ligase NEURL3 suppresses epithelial-mesenchymal transition and metastasis in nasopharyngeal carcinoma by promoting Vimentin degradation**”. We have revised and modified the manuscript in accordance with the editor and the reviewers’ comments and re-submitted the revised manuscript. We all appreciate your support and efforts on our manuscript and look forward to your further decision, and we sincerely hope to have the opportunity to publish this paper in *Journal of Experimental & Clinical Cancer Research*.

A copy of the manuscript indicating where revisions have been made is included in the resubmission. The revisions are indicated using the "Track Changes" function in Word.

**The following is the point-by-point response to the reviewers’ comments and questions.**

**Reviewer #1:**

*The manuscript has delivered a novel idea that the E3 ubiquitin ligase NEURL3 promotes NPC EMT and metastasis by promoting the degradation of Vimentin in a ubiquitin-proteasome pathway. This manuscript has carried out a considerable number of work and provided clear and rigorous arguments, as well as detailed interactions and regulatory mechanisms between NEURL3 and Vimentin in NPC. It is an excellent paper, however, several questions need to be addressed before published.*

*1. This article explores the potential of NEURL3 to interact with Vimentin and mediate its ubiquitination degradation, thereby regulating EMT. Vimentin serves as one of the mesenchymal marker molecules, but I think it is necessary to detect the epithelial marker molecule* *E-cadherin to demonstrate the regulation of EMT.*

**Response:**

We sincerely appreciate your valuable comment. In accordance with your suggestion, we performed Western blot and immunofluorescence assays to assess the expression of E-cadherin after manipulating NEURL3 expression. The results showed that overexpression of NEURL3 obviously increased E-cadherin expression while knockdown of NEURL3 led to a decrease in E-cadherin expression (**Supplementary Fig. 5**), demonstrating that NEURL3 could regulate the EMT process of NPC cells. To address the reviewer’s concern, we have added these results in our revised manuscript (**Page 13, paragraph 2**).

**Supplementary Fig. 5:** E-Cadherin expression in NPC cells after modulation NEURL3 expression.

2. *In Fig. 3e and Fig. 6f, suggest adding corresponding quantization graphs.*

**Response:**

Thank you for your insightful suggestions and comments. We concur with your viewpoint and have added the statistical findings on the filopodia length in each group, which can be found in **Fig. 3e** and **Supplementary Fig. 6b** of our revised manuscript.

**Fig. 3e:** IF staining showing the filopodia length on HONE1, SUNE1, and HK1 cells transfected with HA-NEURL3 plasmid or its empty vector.

**Supplementary Fig. 6b:** The filopodia length on HONE1, SUNE1, and HK1 cells transfected with HA-NEURL3 or its empty vector, in conjunction with Flag-Vimentin or its empty vector.

3. *In Fig. 3a, "P=0.00" and "P<0.00", the P value should be a specific value instead of 0.00.*

**Response:**

We greatly appreciate your valuable suggestions. We have made the necessary revisions of the *p*-values as *p*<0.01 in **Fig. 3a** of our revised manuscript.

**Fig. 3a:** In GSEA analysis using a public RNA-seq dataset (GSE102349), the gene sets associated with cell migration and metastasis pathways were significantly enriched in NPC samples with a low NEURL3 expression.

4. *Fig. 4g need to be marked that "sh1""sh2" is NEURL3.*

**Response:**

Thank you for your suggestion. As per your advice, we have rectified "sh1" and "sh2" in **Fig. 4g** to shNEURL3#1 and shNEURL3#2, respectively.

**Fig. 4g.** Vimentin protein levels in HONE1 and SUNE1 cells transfected with shNEURL3 plasmids or its control vector.

5. *In Fig. 5g-h, there's a slight design flaw, and it might be best to test whether NEURL3 can affect the stability of WT or K97R Vimentin.*

**Response:**

Thanks very much for your valuable suggestions. As you suggest, we have reperformed the experiments, and found that overexpression of NEURL3 enhanced the degradation of Vimentin (WT), but not Vimentin (K97R) when NPC (**Fig. 5g**) and 293T (**Fig. 5h**) cells were treated with CHX. To address the reviewer’s concern, we have replaced the **Fig. 5g-h**, and revised the results of our revised manuscript (**Page 15, paragraph 1**).

**Fig. 5g-h.** Representative images and greyscale analyses of Vimentin protein levels after CHX treatment in SUNE1 (**g**) and 293T (**h**) cells co-transfected with Myc-NEURL3, together with WT Flag-Vimentin or its K97R mutant.

*6. In Fig. 2 and Fig. 7, the IHC need to display clearer high-resolution images.*

**Response:**

We sincerely appreciate your valuable suggestion and have replaced with high-resolution images in both **Fig. 2** and **Fig. 7** of our revised manuscript.

**Reviewer #2:**

*1. For Figure 7i, how to count the number of metastatic tissue? What method do you use？*

**Response:**

Thank you for your comments. We established a lung metastatic colonization model (n=8 per group) by injecting 1×10^6^ SUNE1 cells that stably overexpressed NEURL3 or empty vector via tail vein, and quantified the number of the metastatic tumor nodules as previously described [1]. Briefly, the lung tissues were harvested, paraffin-embedded, and cut into 5 μm sections. One section selected from each lung of the mice were subjected for HE staining and the number of metastatic tumor nodules, typically measuring 1 mm or larger in diameter, in each group was count for statistical analysis. We have described this in the Method section of our revised manuscript (**Page 10, paragraph 1**).

**References:**

1. Zhong L, Liao D, Li J, Liu W, Wang J, Zeng C, et al. Rab22a-NeoF1 fusion protein promotes osteosarcoma lung metastasis through its secretion into exosomes. Signal Transduct Target Ther. 2021; 6(1): 59.
2. *Have you done the NEURL3 gene function in normal cell line vs knock down the gene in normal cell lines in vitro and in vivo?*

**Response:**

Thank you very much for your valuable suggestion. As the normal nasopharynx cell line NP69 cannot form tumors *in vivo*, we performed *in vitro* wound healing and Transwell assays to determine the effect of NEURL3 on NP69 cell migration and invasion by transfecting siNEURL3 or its scramble control. The results showed that knockdown of NEURL3 could enhance the migratory and invasive abilities of NP69 (**Supplementary Fig. 2**).

To address the reviewer’s concern, we have added these results in our revised manuscript (**Page 12, paragraph 2; Supplementary Fig. 2**).

**Supplementary Fig. 2: Knockdown of NEURL3 promotes NP69 cell migration and invasion**

**a.** Relative mRNA and protein levels of NEURL3 in NP69 cells transfected with siNEURL3 or its scramble control. **b.** The migratory abilities of NP69 cells transfected with siNEURL3 or its scramble control determined by wound healing assay. **c.** The migratory and invasive abilities of NP69 cells transfected with siNEURL3 or its scramble control determined by Transwell assay. Data are shown as mean ± SD, and the *p*-values were determined by Student’s t-test (**p* < 0.05).

**Reviewer #3:**

*In the research article entitled "The E3 ligase NEURL3 suppresses epithelial-mesenchymal transition and metastasis in nasopharyngeal carcinoma by promoting Vimentin degradation", the authors reveal a novel mechanism by which NEURL3 inhibits NPC metastasis by promoting Vimentin degradation and provide novel therapeutic targets for NPC patients. This is a well-written paper containing interesting results which merit publication. For the benefit of the reader, however, some important points need clarifying and certain statements require further justification. There are given below.*

**Comments:**

1. *In the first paragraph of Result 1, "NEURL3 is hypermethylated in NPC," the authors propose, "we found the top 20 hypermethylated genes in NPC (n=49) compared with the normal nasopharyngeal tissue (n=49) samples (Fig. 1a). Among them, we selected NEURL3, an E3 ubiquitin ligase, for further analysis." Please further explain why NEURL3 was selected among the top 20 hypermethylated genes instead of other genes for further analysis. What was the basis for choosing NEURL3 for further study?*

**Response:**

Thank you very much for your valuable comments. In our present study, based on an integrated analysis of two genome-wide DNA methylation microarray datasets, we found the top 20 hypermethylated genes in NPC (n=49) compared with normal nasopharyngeal tissue (n=49) samples. Among them, as NEURL3 ranked the top five hypermethylated genes in NPC and it was the most changed E3 ubiquitin ligase, we selected it for further analysis. We have added this in the Result section of our revised manuscript (**Page 10, paragraph 3**).

2. *In Fig 2b, the expression of NEURL3 in CNE1 cell appeared to be higher than in NP69, inconsistent with the corresponding results presented by RT-PCR experiments in Fig 2a. Please verify it again or explain the reason.*

**Response:**

Thanks for your insightful comments. The discrepancy between RNA and protein levels in CNE1 cell line could potentially be attributed to cellular heterogeneity leading to variations in protein expression. Our RT-PCR experiments demonstrated a significant down-regulation of NEURL3 RNA in 9 NPC cell lines compared to NP69 cells. As the reviewer’s suggestion, we have re-performed the western blot experiments and found that NRURL3 protein levels were also downregulated in 9 NPC cell lines (**Supplementary Fig. 2b**).

**Fig. 2b.** Relative protein levels of NEURL3 in NPC cell lines and nasopharyngeal epithelial cell line NP69.

*3.* *In Fig 4f-g, the variation trend of Vimentin protein expression level was not obvious. Please repeat this result and add the densitometry statistical histogram of WB bands.*

**Response:**

As the reviewer suggests, we performed densitometry analysis of Western blot bands in **Fig. 4f**-**g**, and confirmed that overexpression of NEURL3 decreased Vimentin protein levels while knockdown of NEURL3 increased Vimentin protein levels in NPC cells. We have added this in **Fig. 4f** and **Fig. 4g** of our revised manuscript.

**Fig. 4f-g.** Vimentin protein levels in HONE1 and SUNE1 cells transfected with gradient concentrations of HA-tagged NEURL3 plasmid (**f**), as well as shNEURL3 plasmids or its control vector (**g**).

4. *In Fig 7b, please add the growth curve of the mouse tumor and the statistical bar chart of tumor size.*

**Response:**

Thank you very much for your valuable comments. In our present study, we conducted GSEA using an RNA-seq dataset (GSE102349), and found that the gene sets associated with cell migration and metastasis were significantly enriched in NPC samples with a low NEURL3 expression. Furthermore, *in vitro* functional experiments showed that modulation of NEURL3 expression affect the migratory and invasive abilities of NPC cells, but had no effect on NPC cell growth and proliferation. Thus, we did not construct a xenograft tumor growth model to generate growth curves of the mouse tumors and the statistical bar chart of tumor size. In the inguinal lymph node metastasis model, it is very difficult to monitor the growth of the inguinal lymph nodes of mice because of its small size and deep anatomical site. The inguinal lymph nodes were commonly harvested when the mice were killed after 6 weeks for further analysis of the metastatic ratio. Therefore, we could not provide the growth curve of the mouse tumors and the statistical bar chart of tumor size in Fig. 7b.

*5. In Fig 7a, 7d-e and 7g, please show the results of three mice in each group.*

**Response:**

As the reviewer’s suggestions, we have provided the results of three mice in each group in Fig 7a, 7d-e and 7g, and added these pictures in the **Supplementary Fig. 7**.

**Supplementary Fig. 7: NEURL3 inhibits NPC metastasis *in vivo***

**a.** Representative images of the formed footpad tumors and inguinal lymph nodes. **b.** H&E staining showing the infiltration of tumor cells into skin and muscle of footpad tumors (Scale bar, 100 μm). **c.** The infiltration of cancer cells into inguinal lymph nodes as determined by a positive of pan-cytokeratin IHC staining (Scale bar, 2 mm or 20 μm). **g.** Representative images of macroscopic metastatic nodules formed on the lung surfaces of mice in the two groups.

*6. In the discussion section, please add some comments about the limitations of the study.*

**Response:**

Thank you very much for your insightful suggestions. We have added some comments about the limitations of the study “Undoubtedly, this article has its shortcomings. Firstly, our exploration of the mechanism behind NEURL3's inhibition of NPC EMT and metastasis solely focused on the regulation of Vimentin by NEURL3, without delving into potential intermediate molecules. Secondly, due to time, effort, and resource constraints, we did not develop specific methods that up-regulate NEURL3, thus potentially limiting its clinical applicability. These limitations highlight future research directions that we intend to pursue.” In our revised manuscript (**Page 19, paragraph 3; Page 20, paragraph 1**).

*7.* *In the discussion section, please add more discussion about the importance of NEURL3 found in this study and the potential for Vimentin targets to be better used clinically in the future.*

**Response:**

Thank you for your valuable advice. We have added more discussion “Indeed, Vimentin serves as a pro-oncogenic factor that negatively impacts the prognosis of various tumor types. Beyond its involvement in tumor cell EMT and metastasis, Vimentin plays a critical role in tumor angiogenesis, immune infiltration, and immune suppression. Notably, it has been found that a correlation between Vimentin and PD-L1 expression exists in tumor tissues of non-small cell lung carcinoma, and the co-positivity of the two markers is associated with a discernible inclination toward poorer patient prognosis. Thus, we hypothesis that targeted down-regulation of Vimentin expression can not only inhibit NPC metastasis but also potentially enhance the effectiveness of immunotherapy. Immunotherapy holds significant promise in enhancing the clinical effectiveness of NPC treatment, and it is a prominent area of research in the field. As mentioned above, recent evidences have suggested that NEURL3 plays a crucial role in innate immunoregulation. For instance, NEURL3 boosts the host's antiviral response by facilitating K63-linked ubiquitination of IRF7. Therefore, further investigation into the involvement of NEURL3 in the immunoregulation of NPC would shed light on its clinical significance in modulating NPC immunity.” in our revised manuscript (**Page 19, paragraph 2**).

*8. Regarding the newly discovered targets in this study, whether there are further development or research plans, please add it in the discussion.*

**Response:**

Thank for very much for your insightful comments. We have added further research plans “Therefore, further investigation into the involvement of NEURL3 in the immunoregulation of NPC would shed light on its clinical significance in modulating NPC immunity.” in the discussion of our revised manuscript (**Page 19, paragraph 2**).

*9. In general, the conclusion of the discussion section of this article is only a basic summary of the article, which is not enough. Please add more of your own insights into the research so that readers can be excited about the prospects of the study.*

**Response:**

Thank you very much for your suggestions. We have added my own insights “Drawing upon our findings and existing researches, modulating E3 ubiquitin ligase or deubiquitinases to inhibit EMT and metastasis in NPC holds immense promise for enhancing the clinical effectiveness of NPC treatment.” in the conclusion section of our revised manuscript (**Page 20, paragraph 2**).

Your sincerely,

Ying-Qing Li and Qun Zhang
